# Ipsilateral and Contralateral Retinal Ganglion Cells Express Distinct Genes during Decussation at the Optic Chiasm

**DOI:** 10.1523/ENEURO.0169-16.2016

**Published:** 2016-12-02

**Authors:** Qing Wang, Florencia Marcucci, Isadora Cerullo, Carol Mason

**Affiliations:** 1 Doctoral Program in Neurobiology and Behavior; 2 Medical Scientist Training Program; 3 Department of Pathology and Cell Biology; 4 Department of Neuroscience, College of Physicians and Surgeons, Columbia University, New York, NY 10032

**Keywords:** cell specification, contralateral, decussation, ipsilateral, optic chiasm, retinal ganglion cell

## Abstract

The increasing availability of transcriptomic technologies within the last decade has facilitated high-throughput identification of gene expression differences that define distinct cell types as well as the molecular pathways that drive their specification. The retinal projection neurons, retinal ganglion cells (RGCs), can be categorized into distinct morphological and functional subtypes and by the laterality of their projections. Here, we present a method for purifying the sparse population of ipsilaterally projecting RGCs in mouse retina from their contralaterally projecting counterparts during embryonic development through rapid retrograde labeling followed by fluorescence-activated cell sorting. Through microarray analysis, we uncovered the distinct molecular signatures that define and distinguish ipsilateral and contralateral RGCs during the critical period of axonal outgrowth and decussation, with more than 300 genes differentially expressed within these two cell populations. Among the differentially expressed genes confirmed through *in vivo* expression validation, several genes that mark “immaturity” are expressed within postmitotic ipsilateral RGCs. Moreover, at least one complementary pair, Igf1 and Igfbp5, is upregulated in contralateral or ipsilateral RGCs, respectively, and may represent signaling pathways that determine ipsilateral versus contralateral RGC identity. Importantly, the cell cycle regulator cyclin D2 is highly expressed in peripheral ventral retina with a dynamic expression pattern that peaks during the period of ipsilateral RGC production. Thus, the molecular signatures of ipsilateral and contralateral RGCs and the mechanisms that regulate their differentiation are more diverse than previously expected.

## Visual Abstract

**Figure:**
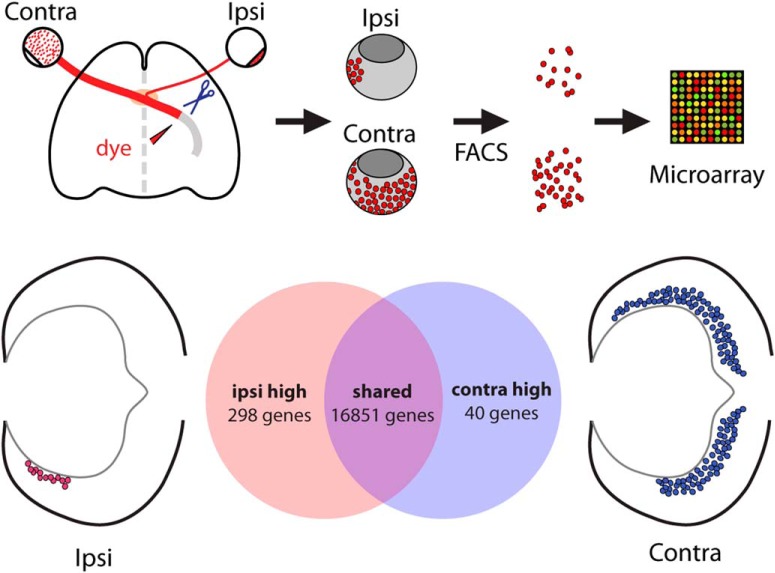

## Significance Statement

This study presents a new method for isolating ipsilaterally and contralaterally projecting retinal ganglion cells (RGCs) via retrograde labeling and fluorescence-activated cell sorting. The subsequent transcriptomic analysis of these purified populations by microarray, followed by *in vivo* expression validation, revealed that ipsilateral RGCs have a distinct set of genes that govern neurogenesis, differentiation, and axon guidance compared with contralateral RGCs. Elucidating these gene programs contributes to our understanding of how decussating systems—in particular, the binocular circuit—are established. This information is critical for directing the appropriate RGC subtype differentiation and axon regeneration for repair after injury.

## Introduction

The vertebrate central nervous system is composed of a complex network of highly diverse neurons defined by distinct molecular signatures that confer their unique properties in morphology, connectivity, and function. The vertebrate retina, with its three cellular layers and six neuronal classes, has been a useful model for studying general principles of neurogenesis and axon guidance. Each class of retinal cells can be further divided into morphologically and functionally distinct subtypes, and recent efforts have identified the molecular programs that establish these differences within neuronal classes, such as amacrine, bipolar, and retinal ganglion cell (RGC) subtypes (Kim et al., 2008; Badea et al., 2009; Kay et al., 2011a, 2011b; Watson et al., 2012; Jiang et al., 2013; Sajgo et al., 2014; Macosko et al., 2015; Osterhout et al., 2015; Sanes and Masland, 2015; Tang et al., 2015; Jin et al., 2015; Rousso et al., 2016; Shekhar et al., 2016). RGCs, as the only projection neurons of the retina, can be additionally distinguished by the laterality of their axonal projection to targets in the thalamus and midbrain. It is this decussation of the retinogeniculate projection that underlies binocular vision.

Two different guidance programs direct the growth of the ipsilateral and contralateral projections at the mouse optic chiasm: EphB1 and EphrinB2 interactions repel ipsilateral axons from the midline, and an NrCAM/PlexinA1 complex reverses an inhibitory Sema6D signal to promote contralateral axon growth through the midline (Williams et al., 2003, 2006b; Kuwajima et al., 2012). Of the known retinal guidance receptors potentially regulated by these transcriptional programs, knockout mouse models show only partial changes in laterality (Williams et al., 2003, 2006a; Erskine et al., 2011; Kuwajima et al., 2012). Moreover, the molecular interactions between transcription factors (e.g., Zic2 and Islet2), downstream effectors (e.g., EphB1, Neuropilin, NrCAM, and PlexinA1), and upstream patterning genes (e.g., Foxd1 and Foxg1) within this genetic network have proven difficult to identify, suggesting the presence of yet-unknown intermediate players that bridge these gaps (Herrera et al., 2003, 2004; Pak et al., 2004; Pratt et al., 2004; Tian et al., 2008; Picker et al., 2009; Carreres et al., 2011; Fotaki et al., 2013; Hernandez-Bejarano et al., 2015). For example, in overexpression studies, Zic2 is more potent than EphB1 in switching RGC projection laterality (Petros et al., 2009b) and thus may regulate additional downstream factors in the uncrossed guidance program. Even less is known about the transcriptional regulators and adhesion molecules that mediate organization of eye-specific RGC axon cohorts in the optic tract and innervation of target regions.

One approach to tackling these questions is to analyze the molecular signatures of ipsilateral and contralateral RGCs to identify genes specific to these two RGC subtypes. Such an approach has proven useful in recent studies of other neuronal subtypes, such as cortical projection neurons (Lodato and Arlotta, 2015), and has been particularly successful in uncovering transcriptional networks that regulate postmitotic cell fate acquisition. An unbiased screen allows for identification of new candidates not previously described in other systems and not ascribed to the retina or RGCs. A challenge to such studies is that ipsilateral RGCs constitute a very small population of cells within the retina [only ∼3–5% of the final RGC number and ∼10% at embryonic day 16.5 (E16.5)]. Thus, the ipsilateral RGC population is particularly sensitive to contamination by other cell types when using anatomical isolation approaches.

Here we present a novel method for purifying embryonic ipsilateral and contralateral RGCs using retrograde labeling of live tissue coupled with fluorescence-activated cell sorting (FACS). Through gene expression profiling of purified ipsilateral and contralateral RGCs during the critical period of axon outgrowth and midline decussation, we have uncovered distinct molecular signatures that define and distinguish these two RGC cohorts during embryonic development. Through subsequent validation of the *in vivo* expression patterns of select candidates, we have identified more than 300 genes that are differentially expressed in ipsilateral and contralateral RGCs. Ipsilateral RGCs are enriched in “early” genes, in particular transcription factors known to be expressed by retinal progenitor cells. Of special interest is the expression of cyclin D2, a cell cycle–related gene highly abundant in ventral retina. In addition, we observed the expression of potential molecular partners in a complementary fashion within ipsilateral and contralateral RGCs. Although several important studies have identified genes that distinguish neuronal subtypes defined by their connectivity or neurotransmitter expression, this is the first attempt at using gene profiling to investigate the differences between two neuronal subtypes distinguished by the laterality of their projection.

## Materials and Methods

### Animals

C57/BL/6J mice were obtained from the Jackson Laboratory (Bar Harbor, ME) and are referred to as wild-type in this study. In Foxd1^lacZ/+^ and Foxd1^lacZ/lacZ^ mice, the lacZ gene was substituted for the coding region of Foxd1 (Hatini et al., 1994). Mice were housed in a barrier facility in a timed-pregnancy colony at Columbia University and exposed only to conditions and procedures that were approved by the Institution Animal Care and Use Committee, protocol numbers AAAG8702 and AAAG9259. Females were checked for vaginal plugs at approximately noon on every weekday. Conception was assumed to take place at midnight, and E0.5 refers to the day on which the vaginal plug was detected.

### Retrograde labeling

Tetramethylrhodamine-conjugated dextran MW3000, anionic, lysine fixable (D-3308; Invitrogen, San Diego, CA) was used to retrogradely label retinal ganglion cells. Pregnant females were anesthetized using ketamine-xylazine (100 and 10 mg/kg, respectively, in 0.9% saline). While the mother was kept alive, each E16.5 embryo was removed from the uterus and decapitated in DMEM/F12 (Invitrogen) buffer over ice. The head was immersed in DMEM, and hard palate and skull base were removed to expose the optic chiasm and tract. The optic tract and surrounding tissue were dried, and a pinch of dextran was applied to the severed optic tract with forceps. Heads were then incubated in pre-oxygenated bubbling artificial CSF at room temperature for 2 hours.

For FACS experiments, retinas were immediately dissected out without fixation and were screened for labeling with rhodamine in the expected domains for ipsilateral and contralateral RGCs. Only retina pairs with robust and specific rhodamine labeling were processed for FACS.

For whole-mount preparations, labeled heads were fixed in 4% PFA in 0.1 m phosphate buffer (PB) at 4°C overnight and rinsed in PBS. Retinas were then dissected and flat-mounted on coverslipped slides with Fluoro-Gel (Electron Microscopy Sciences, Hatfield, PA).

### Fluorescence-activated cell sorting

Dissected ipsilateral or contralateral retinas were digested in papain solution (20 U/mL in Earle’s balanced salt solution, Worthington, Freehold, NJ) followed by repeated trituration in DMEM/F12 + 10% fetal bovine serum + DNaseI. Dissociated cells were washed with PBS, resuspended in PBS + 2% fetal bovine serum, passed through a 0.45-µm cell strainer, and kept on ice before and throughout flow cytometry. Rhodamine-positive neurons, with exclusion of DAPI-stained dead cells, were collected using a BD FACSAria cell sorter in the Columbia University Cancer Research Center core facility.

### RNA extraction

RNA purification was performed immediately after FACS using the Absolutely RNA Nanoprep kit (Stratagene, La Jolla, CA). RNA concentration was determined using the RNA 6000 Pico kit (Agilent Technologies, Santa Clara, CA) with the Agilent 2100 Bioanalyzer and stored at –80°C.

### Microarray preparation and analysis

Amplified cDNA was generated from purified RNA using Ovation Pico WTA System (Nugen, San Carlos, CA) and labeled and fragmented using the Encore Biotin Module (Nugen). For each round of FACS isolation, the same amount of starting RNA extracted from the ipsilateral or contralateral RGC populations was used for cDNA amplification. Labeled cDNA (5 μg) was hybridized on Mouse Genome 430 2.0 Array chips (Affymetrix, Santa Clara, CA), and analyzed using GeneSpring GX11 (Agilent Technologies). Differentially expressed genes were identified from three biological replicates (three independent rounds of retrograde labeling and FACS) by greater than two-fold change and corrected *p* < 0.05, Benjamini–Hochberg. Microarray data was deposited in NCBI’s Gene Expression Omnibus and are accessible through GEO series accession number GSE83461 (https://www.ncbi.nlm.nih.gov/geo/query/acc.cgi?acc=GSE83461).

### Quantitative RT-PCR

cDNA from E14–E16 retina was retrotranscribed from purified RNA using Superscript III Reverse Transcriptase (Invitrogen). Quantitative PCR (qPCR) was performed using a Stratagene MX3000 with the SYBR Green PCR Kit (Applied Biosystems, Foster City, CA). Transcript levels were normalized to that of HPRT. qPCR-specific primers (Table 1) were designed using the Primer3 program.

**Table 1.** Primers used in qRT-PCR and for generating *in situ* hybridization probes.

Gene	Forward Primer	Reverse Primer	Amplicon Size (bp)
Used in RT-PCR			
*Zic2*	GCATGTCCACACCTCAGATAAG	ATGGACCTTCATGTGCTTCC	324
*Slc6a4 (SERT)*	GCTGAGATGAGGAACGAAGAC	GAGGAAGAAGATGATGGCAAAG	1240
*Gja1*	TACCCAACAGCAGCAGACTTT	AAATGAAGAGCACCGACAGC	240
*Klf4*	CCCAACTACCCTCCTTTCCT	AGGTTTCTCGCCTGTGTGAGT	2175
*Lhx2*	GCGAATACCCAGCACACTTT	TGTTCAGCATCGTTCTCGTT	235
*Otx2*	GTTCCGTCACTCCAAATCTACC	GTCCTCTCCCTTCGCTGTTT	248
*Sox2*	GTTACCTCTTCCTCCCACTCCA	CTTCTCCAGTTCGCAGTCCA	216
*Sparc*	GACTCTTCCTGCCACTTCTTTG	AGGTTGTTGCCCTCATCTCTCT	1454
*Zfp36*	CCCATCTTCAATCGTATCTCTG	CTGTCAACTGTCTCCCTCAAAC	1765
*Zic1*	GCAAGATGTGCGATAAGTCC	GTGGTCGGGTTGTCTGTTGT	744
*Sema3e*	CCAACTCCTCCTTTGTGTCC	TCATCTCGGTCTTCGTTATCAG	2570
*Tbx20*	ATTGAGAGGGAGAGTGTGGAGA	ACGATACCCAGGAACTGAGAGA	1338
*Hprt*	AGCAGGTGTTCTAGTCCTGTGG	ACGCAGCAACTGACATTTCTAA	101
Used for generating ISH probes			
*Ccnd2*	TCCTGTTTGCCTTCCTTGGAGCCT	ATATGACGGGCTCTGCTTTCCCGT	566
*Fgf12*	GGAGACTTAGGGAACTCGCTGGCA	CACGACCCAAACCCACCCACAAAA	500
*Igf1*	AGCAGATAGAGCCTGCGCAATGGA	CGGGGACTTCTGAGTCTTGGGCAT	551
*Igfbp5*	CTCCAACCCGGAACATGGAGCAAC	GCCCACATAAGGGACAGAGGCTCA	508
*Sox2*	ACAAGGGAATTGGGAGGGGTGCAA	AACCCAGCAAGAACCCTTTCCTCG	504
*Tbx20*	TGGGTGAGCTGACGAGTCTGGATG	TATGATGTGCACCCGTGGCTGGTA	536
*Zic2*	AACCCATCGAGGGCACCTTAGGAT	AGGGAGACTTTGGCACGGCTCATA	600

### Generation of *in situ* hybridization probes

Plasmids for making *in situ* hybridization (ISH) probes were generated for select microarray candidates. cDNA sequences were obtained from NCBI, and PCR primers for generating ∼500-bp riboprobe sequences were selected using Primer-BLAST. 3′ sequences of the gene were targeted if possible (Table 5). Template cDNA was synthesized from RNA purified from E14–E16 retina using Superscript III Reverse Transcriptase (Invitrogen), and gene product was generated with targeted oligos and Platinum Pfx polymerase (Invitrogen). PCR product was purified using QIAquick PCR Purification kit (Qiagen, Hilden, Germany), inserted into pCR-Blunt II-TOPO vector using Zero Blunt Cloning kit (Invitrogen), and transformed into TOP10 or DH5α cells. Purified plasmids were linearized using restriction enzyme with overnight incubation at 37°C. Riboprobes were synthesized using the Digoxigenin RNA labeling mix (Roche, Basel, Switzerland). The following probe plasmids were obtained from other laboratories: Math5 (L. Gan, University of Rochester), Sema3e (A. Kolodkin, Johns Hopkins University), and Tbx20 (G. Papaioannou, Columbia University).

### *In situ* hybridization of retinal sections

Embryos were collected at E13.5, E15.5, or E16.5 in PBS on ice. Heads were decapitated in PBS over ice, fixed in 4% paraformaldehyde (PFA) in 0.1 m PB at 4°C overnight, rinsed in PBS at least three times for a minimum of 1 h total at 4°C, and cryoprotected in 30% sucrose in 0.1 m PBS for 1–2 nights at 4°C. Coronal sections (14-µm) were collected through the retina and immediately processed for ISH (or stored at –80°C) using a protocol adapted from Schaeren-Wiemers and Gerfin-Moser (1993). After ISH, tissues were fixed for 30 min in 4% PFA, washed with PBS, and processed for immunostaining with Zic2 and Islet1/2 antibodies.

### Antibodies

The following primary antibodies were used: rabbit anti-Zic2 (RRID:AB_2315623, gift of Stephen Brown, 1:10,000), mouse anti-Islet1/2 (cat. # 39.4D5, RRID:AB_528173, gift of Thomas Jessell, 1:50; this antibody was raised against Islet1 but also reacts with Islet2), rabbit anti-cyclin D2 (Santa Cruz Biotechnology, Santa Cruz, CA, cat. # sc-452, RRID:AB_627350, 1:1000), rat anti-cyclin D2 (Santa Cruz Biotechnology, cat. # sc-593 RRID:AB_2070794, 1:50), goat anti-Brn3 (Santa Cruz Biotechnology, cat. # sc-6026 RRID:AB_673441, 1:200), and mouse anti-Brn3a (EMD Millipore, Billerica, MA, cat. # MAB1585 RRID:AB_94166, 1:200).

The following secondary antibodies were used: donkey anti-rabbit Alexa Fluor488 (Invitrogen, 1:400), donkey anti-mouse Alexa Fluor 488 (Invitrogen, 1:400), donkey anti-goat Alexa Fluor 488 (Invitrogen, 1:400), donkey anti-mouse Cy3 (Jackson Immunoresearch, West Grove, PA, 1:500), donkey anti-rabbit Cy3 (Jackson Immunoresearch, 1:500), donkey anti-goat Cy5 (Jackson Immunoresearch, 1:200), and donkey anti-rat Cy3 (Jackson Immunoresearch, 1:500).

### Immunostaining of retinal sections

Embryos were collected at the age of interest in PBS on ice. Heads were decapitated in PBS over ice, fixed in 4% PFA in 0.1 m PB for 1 h at 4°C, rinsed in PBS at least three times, washed in PBS for a minimum of 1 h at 4°C, and cryoprotected in 10% sucrose in 0.1 m PBS for 1–2 nights at 4°C. Heads were embedded in optimal cutting temperature compound over crushed dry ice and stored at –80°C. 12 μm coronal sections were collected for cyclin D2 immunostaining; 16- to 20-µm coronal sections were collected for all other stainings. For immunohistochemistry (IHC), slides were blocked in 10% normal goat serum (NGS) and 0.2% Triton X-100 in PBS for 1 h, incubated with primary antibody in 1% NGS and 0.2% Triton X-100 in PBS overnight at 4°C, washed three times for 20 min in PBS at room temperature, incubated in secondary antibody in 1% NGS and 0.2% Triton X-100 in PBS overnight at 4°C, and washed in PBS three times for 20 min each. Coverslips were mounted on slides with Fluoro-Gel.

### Imaging

ISH and whole-mount preparations (anterograde and retrograde labeling) were taken by Axiovision software with an Axiophot camera connected to a Zeiss Axioplan2 microscope. IHC preparations were imaged using a Zeiss AxioImager M2 microscope equipped with Apotome, AxioCam MRm camera, and Neurolucida software (V10.40, MBF Bioscience, Williston, VT).

## Results

### Purification of ipsilaterally and contralaterally projecting RGCs

**Figure 1.**
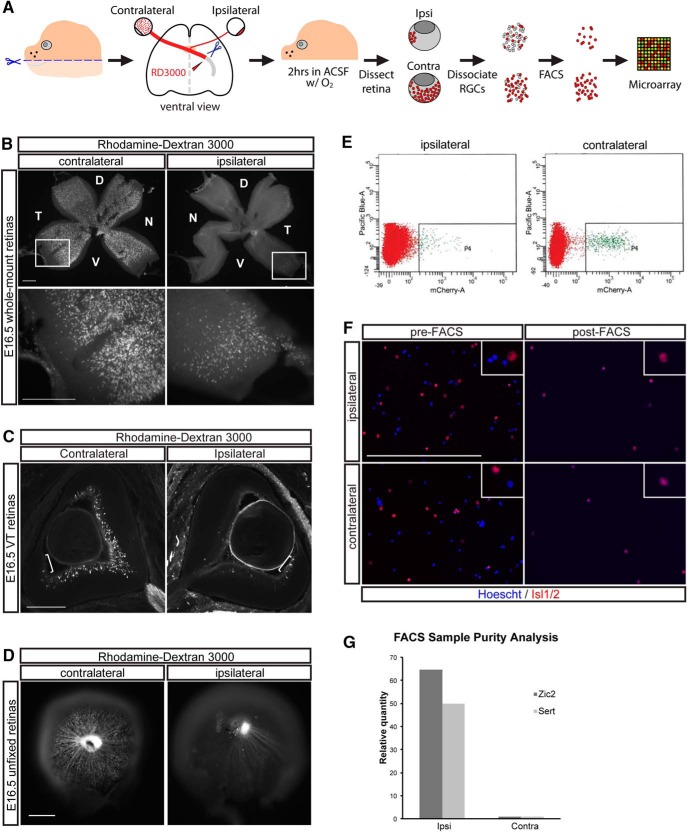
FACS purification of retrogradely labeled mouse retinal ganglion cells at E16.5. ***A***, Schematic of retrograde labeling and cell purification methods for microarray analysis. Diagram of the ventral view of embryonic brain depicts application of rhodamine dextran 3000 MW dye (RD3000) to a unilateral transected optic tract. ***B***, Whole-mount preparations of E16.5 retina. RD3000 fully labels axons and cell bodies of E16.5 RGCs within 2 hours of incubation after dye application to the optic tract. Extra-VT RGCs are labeled in retina contralateral to labeled optic tract and VT RGCs are labeled in the ipsilateral retina. ***C***, Coronal vibratome sections of E16.5 retina retrogradely labeled with RD3000 show specific labeling of contralateral and ipsilateral RGCs in their respective retinal domains (VT domain marked with white bracket). ***D***, Fresh E16.5 retinas are screened for appropriate RD3000 labeling of ipsilateral and contralateral RGCs before FACS. ***E***, FACS purification of ipsilateral and contralateral RGC populations retrogradely labeled with RD3000 with DAPI exclusion of nonviable cells. ∼3000 ipsilateral and ∼20,000 contralateral RGCs (P4 gate) are purified from two litters of E16.5 embryos. ***F***, Cells purified by FACS are enriched in RGC marker Islet1/2 compared with presorting. ***G***, Zic2 and SERT are enriched in the ipsilateral RGC cell population isolated by FACS compared with the contralateral RGCs. D, dorsal, V, ventral, N, nasal, T, temporal. Scale bars, 250 μm.

To identify genes that distinguish ipsilateral and contralateral RGCs during early development through gene expression profiling, pure populations of these two RGC subpopulations from mouse embryonic eyes are needed. To accomplish this, we devised a method of combining retrograde axonal tracing from the optic tract with FACS. In this procedure, the optic tracts are accessed by removal of the palate and exposure of the ventral brain in an isolated head preparation. We then cut the optic tract unilaterally and applied to the cut site a fluorophore-conjugated dextran, which is subsequently transported retrogradely to RGC somas within the ipsilateral or contralateral retina (Fig. 1*A*). Traditional retrograde labeling approaches for the visual system require long overnight incubations for dye transport. We surveyed a number of commonly used retrograde tracers (including cholera toxin B and dextrans of various molecular weights) and devised a protocol for retrogradely labeling RGCs using rhodamine-dextran 3000 within 2 hours of dye application. These conditions are optimal for preserving tissue health for subsequent cell sorting experiments. As expected, the RGCs labeled within the ipsilateral retina were located in the ventrotemporal (VT) peripheral crescent, whereas extra-VT RGCs were labeled in the contralateral retina as seen in whole mounts (Fig. 1*B*) and cross-sections (Fig. 1*C*). The fluorescence signal within labeled retinas from two E16.5 litters (12–15 embryos total) was examined, and only retinal pairs with specific and sufficient RGC labeling were used for cell sorting (Fig. 1*D*).

Pooled ipsilateral and contralateral retinas (8–10 each) were dissociated to obtain a single-cell suspension, and rhodamine-dextran–positive cells were then isolated by FACS. Two litters of retrogradely labeled embryos yielded approximately 3000 FACS events for the ipsilateral population and 20,000 for the contralateral (Fig. 1*E*). Plating of cells before and after FACS shows enrichment in cells that stain positively for Islet1/2, a marker of differentiated RGCs (Fig. 1*F*). RGCs were immediately lysed for RNA isolation after FACS, with total time from removal of embryo for retrograde labeling to post-FACS RNA isolation being 8–9 hours. Subsequent qPCR analysis showed that rhodamine-labeled RGCs from ipsilateral retinas express high levels of Zic2 and SERT compared with those from contralateral retinas (Fig. 1*G*). Thus, rapid retrograde labeling from the optic tract *ex vivo* followed by FACS is a clean and effective way for purifying ipsilateral and contralateral RGCs during development.

### Expression profiling of ipsilateral and contralateral RGCs confirms differential expression of known ipsilateral and contralateral markers and reveals novel differences

We next conducted expression profiling of ipsilateral and contralateral RGC populations purified using the above methods. Because of the small number of ipsilateral RGCs isolated with this method, cDNA prepared from FACS-isolated ipsilateral and contralateral RGCs was amplified before gene chip hybridization. The three biological replicates from independent rounds of retrograde labeling and FACS that showed the most robust enrichment in Zic2 expression in ipsilateral RGCs by qPCR were selected for microarray analysis. For each round of FACS, paired ipsilateral and contralateral RGCs underwent the same treatment conditions and sample preparation methods.

**Figure 2.**
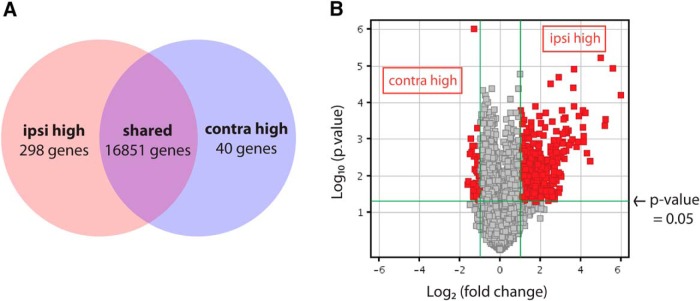
Microarray analysis of ipsilateral and contralateral RGCs purified at E16 reveals distinct expression profiles. ***A***, Microarray analysis reveals 298 and 40 unique genes at least two-fold increased in ipsilateral or contralateral RGCs, respectively (*p* ≤0.05, Benjamini–Hochberg correction). ***B***, Distribution of differentially expressed genes shows that the majority are upregulated in ipsilateral RGCs.

Gene ontology analysis of microarray results (GEO series accession number GSE83461) revealed that many differentially expressed genes are involved in developmental processes, including regulation of gene expression, cell proliferation, cell cycle progression, and cell differentiation (Table 4). Most genes were expressed at similar levels in ipsilateral and contralateral RGC samples, including the pan-RGC transcription factors Brn3b and Islet1 (Pan et al., 2008). However, 338 genes were differentially expressed in the two populations by two-fold or more (corrected *p*-value <0.05, Benjamini–Hochberg; Fig. 2*A*, *B*, Table 5) and included genes known to be enriched in ipsilateral RGCs such as Zic2 (5.78-fold) and SERT (4.49-fold), as well as contralateral RGC marker Brn3a (2.20-fold). The other known contralateral RGC marker, Islet2, was elevated by only 1.5-fold and did not make the two-fold cutoff; however, this may be because Islet2 is expressed in only a subset (∼33%) of contralateral RGCs (Pak et al., 2004).

The microarray gene list also included genes not previously characterized with regard to their differential expression in RGC subpopulations, most of which were elevated in ipsilateral RGCs (Fig. 2*A*). In total, 298 genes (404 probe sets) were found to be enriched in ipsilateral RGCs and 40 genes (47 probe sets) in contralateral RGCs, using a two-fold cutoff (Fig. 2, Table 5), suggesting that an extensive genetic program is activated to generate ipsilateral RGCs. An alternative explanation for these findings is that because ipsilateral RGCs reside in a more spatially restricted domain within the retina, some of these genes may have region-specific expression patterns. Moreover, contralateral RGCs represent a much larger cellular population than ipsilateral RGCs in mice, and subsets of contralateral RGCs themselves may have diverse gene expression profiles. Thus, genes that are expressed exclusively in a subset of contralateral RGCs may have been thus missed by our analysis, as in the case of Islet2.

For validation of the microarray, we analyzed the expression levels of 11 new gene candidates (nine ipsilateral high and two contralateral high) in FACS-derived RGCs by quantitative RT-PCR. Of these 11 genes, 10 (all but Otx2) were enriched in the RGC subpopulation as seen by gene profiling, demonstrating interplatform reproducibility of the gene expression detection by microarray (Table 2). In summary, gene profiling of purified ipsilateral and contralateral RGCs at E16.5 revealed that these two RGC subsets are defined by unique molecular signatures and provided an extensive list of candidate genes that may be differentially expressed in these two cell populations.

**Table 2.** qRT-PCR validation of select microarray candidates shows interplatform reproducibility.

		Microarray		qPCR
Gene symbol	Gene name	FC	*p*-value	FC
High in ipsilateral RGCs				
Gja1	Gap junction protein, alpha 1 (connexin 43)	17.60	0.001	>500
Klf4	Kruppel–like factor 4	7.38	0.002	455
Lhx2	LIM homeobox protein 2	4.72	0.009	2.37
*Otx2*	*Orthodenticle homolog 2*	*5.02*	*0.021*	*0.651*
**Slc6a4**	**Serotonin transporter (SERT)**	**4.49**	**0.004**	**49.8**
Sox2	SRY–box containing gene 2	7.47	0.029	3.81
Sparc	Secreted acidic cysteine rich glycoprotein	12.47	0.000	6.02
Zfp36	Zinc finger protein 36	8.28	0.002	31.2
Zic1	Zinc finger protein of the cerebellum 1	5.67	0.002	6.49
**Zic2**	**Zinc finger protein of the cerebellum 2**	**5.78**	**0.016**	**64.7**
High in contralateral RGCs				
Sema3e	Semaphorin 3E	–2.20	0.003	–2.52
Tbx20	T–box 20	–2.15	0.001	–42.5

Of these 11 genes, all but Otx2 (italicized) were enriched in the RGC subpopulation as seen by gene profiling, demonstrating interplatform reproducibility of the gene expression detection by microarray. Genes previously known to be differentially expressed in ipsilateral and contralateral RGCs are bolded (Zic2, SERT).

### Selection of differentially expressed gene candidates for *in vivo* expression validation

**Figure 3.**
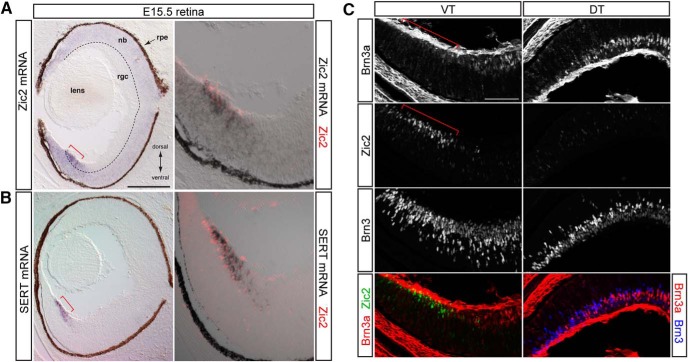
Expression patterns of known ipsilateral and contralateral RGC genes. Combined ISH and IHC analysis at E15.5 shows colocalization of Zic2 protein expression with Zic2 (***A***) and SERT (***B***) mRNA in ipsilateral RGCs in the VT retina. ***C***, In contrast, contralateral RGC marker Brn3a shows complementary expression to Zic2 in immunostained sections in which all RGCs are labeled with a pan-Brn3 antibody. These patterns of expression were used as standards for expression analysis of microarray gene candidates. rpe, retinal pigment epithelium; nb, neuroblast layer; rgc, retinal ganglion cell layer; DT, dorsotemporal retina; VT, ventrotemporal retina. Scale bars, 250 μm (A and B) and 100 μm (C).

To validate the expression of candidate genes *in vivo*, we used ISH and IHC, which provide information regarding the spatiotemporal pattern of expression as well as colocalization with known ipsilateral or contralateral RGC markers, such as Zic2, SERT, and Brn3a (Fig. 3). The primary purpose of this microarray screen was to identify additional regulators of axon guidance at the midline and target, including guidance molecules and transcription factors, other genes that functionally distinguish ipsilateral and contralateral RGCs, and finally, genes that specify ipsilateral and contralateral RGC fate. Thus, of the 339 differentially expressed genes, we focused on five groups: transcription factors involved in neuronal development (e.g. Sox2, Lhx2, Math5, Tbx20), signaling pathways prominent during development (e.g. Igf1, Igfbp5, Fgf12, Ptch1), axon guidance–related genes (e.g. Sema3e, Sema4d, Sema5b, Sema7a), cell cycle regulators (e.g., Ccnd1, Ccnd2), and other cell-surface or secreted molecules that may be related to cell differentiation (e.g., Gja1, Sparc, Zip6/Liv-1, Napb). Representative genes of these groups are shown in Table 3.

**Table 3.** Representative genes that are differentially expressed in ipsi- and contralateral RGCs.

Function	Gene symbol	Gene name	Fold change	*p*-value
High in ipsilateral RGCs				
Regulation of transcription	Atoh7	Atonal homolog 7 (Math5)	3.87	0.010
	Gli3	GLI-Kruppel family member GLI3	3.19	0.015
	Klf4	Kruppel-like factor 4	7.38	0.002
	Lhx2	LIM homeobox protein 2	4.72	0.009
	Neurod4	Neurogenic differentiation 4	6.00	0.013
	Otx2	Orthodenticle homolog 2	5.02	0.021
	Sox2	SRY-box containing gene 2	7.47	0.029
	Sox9	SRY-box containing gene 9	5.69	0.003
	Zic1	Zinc finger protein of the cerebellum 1	5.67	0.002
	**Zic2**	**Zinc finger protein of the cerebellum 2**	**5.78**	**0.016**
Developmental signaling	Fzd5	Frizzled homolog 5	5.04	0.024
	Notch2	Notch gene homolog 2	6.02	0.031
	Tgfb2	Transforming growth factor, beta 2	6.36	0.005
Axon guidance	Sema5b	Semaphorin 5B	4.11	0.007
Cell cycle control	Ccnd2	Cyclin D2	8.17	0.002
Other cellular functions	Gja1	Gap junction protein, alpha 1 (connexin 43)	17.60	0.001
	Slc1a3	Glial high affinity glutamate transporter	9.47	0.002
	Slc2a1	Facilitated glucose transporter	3.19	0.016
	**Slc6a4**	**Serotonin transporter (Sert)**	**4.49**	**0.004**
	Sparc	Secreted acidic cysteine rich glycoprotein	12.47	0.000
High in contralateral RGCs				
Regulation of transcription	**Pou4f1**	**POU domain, class 4, transcription factor 1 (Brn3a)**	**2.20**	**0.001**
	Tbx20	T-box 20	2.15	0.001
Signaling	Bmper	BMP-binding endothelial regulator	2.83	0.002
Axon guidance	Sema3e	Semaphorin 3E	2.20	0.003
	Sema7a	Semaphorin 7A	2.03	0.022

Genes previously known to be differentially expressed in ipsilateral and contralateral RGCs are bolded (Zic2, SERT, Brn3a).

**Table 4.** Gene ontology analysis of differentially expressed genes.

Gene ontology term	Corrected *p*-value	Count in selection	% Count in selection
Cell cycle process	6.91E–16	26	9.737827
Cell cycle	1.56E–15	46	17.228464
Multicellular organismal development	6.29E–14	75	28.089888
Developmental process	8.59E–14	81	30.337078
Anatomical structure development	1.57E–13	55	20.59925
System development	1.57E–13	51	19.101124
Cellular component organization	3.05E–13	48	17.977528
Cellular component organization at cellular level	4.22E–13	48	17.977528
Cellular component organization or biogenesis	8.20E–12	48	17.977528
Cell cycle phase	9.66E–12	22	8.2397
Cellular component organization or biogenesis at cellular level	1.33E–11	48	17.977528
Multicellular organismal process	4.19E–11	75	28.089888
Cell division	1.10E–10	28	10.486892
Nervous system development	3.68E–10	33	12.35955
M phase	3.74E–10	22	8.2397
Organelle organization	1.32E–08	39	14.606742
Cellular developmental process	1.33E–08	41	15.355805
Cell differentiation	1.33E–08	41	15.355805
Organ development	1.77E–08	29	10.8614235
Mitotic cell cycle	2.37E–08	22	8.2397
Cellular component assembly at cellular level	6.85E–08	20	7.490637
Positive regulation of cellular process	4.30E–07	40	14.981274
Positive regulation of biological process	5.22E–07	40	14.981274
M phase of mitotic cell cycle	6.40E–07	22	8.2397
DNA binding	9.46E–07	73	27.340824
Cellular component assembly	9.46E–07	20	7.490637
Mitosis	2.06E–06	21	7.8651686
Nuclear division	2.06E–06	21	7.8651686
Organelle fission	3.32E–06	21	7.8651686
Anatomical structure morphogenesis	5.73E–06	16	5.9925094
Embryo development	5.94E–06	10	3.7453184
Negative regulation of biological process	8.17E–06	19	7.116105
Neurogenesis	1.17E–05	15	5.6179776
Organ morphogenesis	2.01E–05	11	4.11985
Regulation of cell proliferation	2.10E–05	27	10.11236
Cellular macromolecular complex assembly	2.11E–05	20	7.490637
Central nervous system development	2.34E–05	10	3.7453184
Negative regulation of cellular process	2.34E–05	19	7.116105
Cellular macromolecular complex subunit organization	2.52E–05	20	7.490637
Regulation of developmental process	4.17E–05	5	1.8726592
Protein polymerization	5.46E–05	9	3.3707864
Cytoplasmic microtubule	5.50E–05	7	2.621723
DNA-dependent DNA replication initiation	6.27E–05	6	2.247191
Cell fate commitment	6.27E–05	9	3.3707864
Pattern specification process	6.27E–05	14	5.243446
Response to external stimulus	6.69E–05	1	0.37453184
Cellular component biogenesis	6.70E–05	20	7.490637
Tube morphogenesis	1.01E–04	1	0.37453184
DNA replication	1.01E–04	15	5.6179776
Regulation of cell cycle	1.19E–04	11	4.11985
Neural tube development	1.19E–04	6	2.247191
Chromosome	1.28E–04	14	5.243446
Chordate embryonic development	1.37E–04	7	2.621723
Embryo development ending in birth or egg hatching	1.55E–04	7	2.621723
Negative regulation of cell proliferation	1.58E–04	12	4.494382
Generation of neurons	1.64E–04	6	2.247191
Regulation of cell differentiation	1.64E–04	3	1.1235955
Tube development	1.90E–04	2	0.7490637
Tissue morphogenesis	2.50E–04	3	1.1235955
Microtubule-based process	2.63E–04	12	4.494382
Regulation of macromolecule metabolic process	2.64E–04	75	28.089888
Biological regulation	3.12E–04	100	37.453182
Macromolecular complex assembly	3.32E–04	20	7.490637
Macromolecular complex subunit organization	3.32E–04	20	7.490637
Brain development	4.09E–04	6	2.247191
Nucleus	4.09E–04	137	51.31086
Gliogenesis	4.52E–04	4	1.4981273
Regulation of biological process	4.68E–04	100	37.453182
Microtubule cytoskeleton	4.97E–04	16	5.9925094
Regulation of metabolic process	6.17E–04	76	28.46442
Sensory organ development	7.76E–04	4	1.4981273
Negative regulation of cell differentiation	8.23E–04	3	1.1235955
Response to stimulus	8.78E–04	9	3.3707864
Cell proliferation	9.19E–04	13	4.8689137
DNA conformation change	9.32E–04	11	4.11985
Embryonic morphogenesis	0.001010836	3	1.1235955
DNA packaging	0.001010836	11	4.11985
Protein binding	0.001034775	156	58.426968
Microtubule-based movement	0.00124575	12	4.494382
Biological process	0.001371541	203	76.02996
Actin filament organization	0.001405562	5	1.8726592
Regulation of cellular metabolic process	0.001470854	68	25.468164
Camera-type eye development	0.001559894	3	1.1235955
Neuron differentiation	0.001583902	6	2.247191
Nucleosome assembly	0.001716816	11	4.11985
Cell development	0.001716816	3	1.1235955
Negative regulation of developmental process	0.001716816	3	1.1235955
Regulation of epithelial cell proliferation	0.001716816	1	0.37453184
Cytoskeleton organization	0.001748142	7	2.621723
Leukocyte migration	0.001748142	2	0.7490637
Positive regulation of transcription from RNA polymerase II promoter	0.001799044	23	8.614232
Embryonic organ development	0.001913692	2	0.7490637
Chromatin assembly	0.001913692	11	4.11985
Positive regulation of macromolecule metabolic process	0.002041641	27	10.11236
Protein-DNA complex assembly	0.002041641	11	4.11985
Nucleosome organization	0.002041641	11	4.11985
Viral infectious cycle	0.00230068	4	1.4981273
Eye development	0.002356627	4	1.4981273
Protein-DNA complex subunit organization	0.002392596	11	4.11985
Cell fate specification	0.003150978	5	1.8726592
Gamma-aminobutyric acid metabolic process	0.003164838	4	1.4981273
Negative regulation of cell cycle	0.003394165	5	1.8726592
Cellular component	0.003511103	185	69.28839
Heart development	0.003523669	11	4.11985
Regulation of cellular process	0.003547314	93	34.83146
Response to stress	0.003999738	7	2.621723
Regulation of gene expression	0.004208025	75	28.089888
Cellular process	0.005303409	121	45.31835
Positive regulation of transcription, DNA-dependent	0.005370182	24	8.988764
Regionalization	0.005463669	8	2.9962547
Positive regulation of cell proliferation	0.005663401	14	5.243446
Response to wounding	0.005722695	4	1.4981273
Positive regulation of RNA metabolic process	0.005722695	24	8.988764
Positive regulation of metabolic process	0.007105737	27	10.11236
Glial cell differentiation	0.007105737	3	1.1235955
Hindbrain development	0.007105737	2	0.7490637
*O*-fucosylpeptide 3-beta-N-acetylglucosaminyltransferase activity	0.007105737	3	1.1235955
Dorsal/ventral pattern formation	0.008772324	8	2.9962547
Embryonic organ morphogenesis	0.008772324	1	0.37453184
Epidermis morphogenesis	0.008772324	1	0.37453184
Cytoskeletal part	0.009421547	15	5.6179776
Tissue development	0.009551329	5	1.8726592
Cellular component movement	0.009551329	6	2.247191

**Table 5.** Genes differentially expressed in ipsilateral versus contralateral RGCs.

Probe set ID	Gene symbol	Fold change	*p*-value	Regulation	Entrez gene ID
1437726_x_at	C1qb	63.10	0.0001	Up	12260
1419872_at	Csf1r	47.49	0.0000	Up	12978
1448591_at	Ctss	36.91	0.0003	Up	13040
1434366_x_at	C1qb	36.62	0.0004	Up	12260
1452141_a_at	Sepp1	31.76	0.0000	Up	20363
1436905_x_at	Laptm5	21.92	0.0040	Up	16792
1448617_at	Cd53	19.88	0.0023	Up	12508
1449401_at	C1qc	18.75	0.0002	Up	12262
1437992_x_at	Gja1	17.61	0.0008	Up	14609
1450792_at	Tyrobp	17.54	0.0012	Up	22177
1427683_at	Egr2	17.35	0.0002	Up	13654
1419561_at	Ccl3	17.30	0.0005	Up	20302
1422903_at	Ly86	14.98	0.0002	Up	17084
1418204_s_at	Aif1	14.64	0.0011	Up	11629
1415800_at	Gja1	13.85	0.0015	Up	14609
1427076_at	Mpeg1	12.73	0.0001	Up	17476
1448392_at	Sparc	12.47	0.0000	Up	20692
1416589_at	Sparc	12.24	0.0000	Up	20692
1437983_at	Sall1	11.81	0.0010	Up	58198
1438945_x_at	Gja1	11.25	0.0012	Up	14609
1433933_s_at	Slco2b1	10.56	0.0014	Up	101488
1419873_s_at	Csf1r	10.41	0.0006	Up	12978
1415931_at	Igf2	9.71	0.0006	Up	16002
1427682_a_at	Egr2	9.51	0.0005	Up	13654
1426340_at	Slc1a3	9.47	0.0020	Up	20512
1431724_a_at	P2ry12	9.41	0.0003	Up	70839
1431057_a_at	Prss23	9.39	0.0006	Up	76453
1417457_at	Cks2	9.15	0.0032	Up	66197
1460180_at	Hexb	8.92	0.0003	Up	15212
1448891_at	Fcrls	8.72	0.0021	Up	80891
1438658_a_at	S1pr3	8.58	0.0013	Up	13610
1423452_at	Stk17b	8.53	0.0012	Up	98267
1456219_at	LOC100045988	8.47	0.0007	Up	100045988
1452519_a_at	Zfp36	8.28	0.0002	Up	22695
1434745_at	Ccnd2	8.17	0.0012	Up	12444
1439067_at	Lair1	8.14	0.0101	Up	52855
1417496_at	Cp	8.12	0.0030	Up	12870
1422313_a_at	Igfbp5	7.92	0.0087	Up	16011
1416124_at	Ccnd2	7.73	0.0221	Up	12444
1424118_a_at	Spc25	7.67	0.0029	Up	66442
1456140_at	LOC100045988	7.47	0.0011	Up	100045988
1416967_at	Sox2	7.46	0.0287	Up	20674
1417394_at	Klf4	7.38	0.0000	Up	16600
1439902_at	C5ar1	7.38	0.0078	Up	12273
1451318_a_at	LOC676654///Lyn	7.24	0.0024	Up	17096///676654
1426454_at	Arhgdib	7.17	0.0030	Up	11857
1437874_s_at	Hexb	7.14	0.0012	Up	15212
1450644_at	Zfp36l1	7.01	0.0043	Up	12192
1452031_at	Slc1a3	6.91	0.0006	Up	20512
1448160_at	Lcp1	6.89	0.0014	Up	18826
1420029_at	Mcm3	6.89	0.0129	Up	17215
1455333_at	Tns3	6.83	0.0022	Up	319939
1429428_at	Tcf7l2	6.76	0.0019	Up	21416
1439040_at	Cenpe	6.53	0.0288	Up	229841
1436174_at	Atad2	6.50	0.0052	Up	70472
1450923_at	Tgfb2	6.36	0.0055	Up	21808
1416855_at	Gas1	6.34	0.0081	Up	14451
1422134_at	Fosb	6.32	0.0163	Up	14282
1450779_at	Fabp7	6.32	0.0088	Up	12140
1446742_at	Nfia	6.31	0.0075	Up	18027
1435436_at	Epas1	6.31	0.0010	Up	13819
1419309_at	Pdpn	6.23	0.0019	Up	14726
1436756_x_at	Hadh	6.21	0.0276	Up	15107
1426910_at	Pawr	6.12	0.0328	Up	114774
1453748_a_at	Kif23	6.06	0.0268	Up	71819
1455556_at	Notch2	6.02	0.0307	Up	18129
1421492_at	Ptgds2	6.02	0.0003	Up	54486
1450567_a_at	Col2a1	6.00	0.0027	Up	12824
1436694_s_at	Neurod4	6.00	0.0133	Up	11923
1456060_at	Maf	5.95	0.0041	Up	17132
1450922_a_at	Tgfb2	5.93	0.0326	Up	21808
1445740_at		5.91	0.0364	Up	
1421301_at	Zic2	5.78	0.0164	Up	22772
1458447_at	Cenpf	5.76	0.0249	Up	108000
1448475_at	Olfml3	5.71	0.0000	Up	99543
1451538_at	Sox9	5.69	0.0034	Up	20682
1441520_at	Aspm	5.68	0.0242	Up	12316
1417458_s_at	Cks2	5.67	0.0016	Up	66197
1423477_at	Zic1	5.67	0.0022	Up	22771
1422814_at	Aspm	5.64	0.0175	Up	12316
1422694_at	Ttyh1	5.46	0.0038	Up	57776
1416816_at	Nek7	5.42	0.0113	Up	59125
1436363_a_at	Nfix	5.27	0.0036	Up	18032
1434437_x_at	Rrm2	5.15	0.0108	Up	20135
1452428_a_at	B2m	5.11	0.0036	Up	12010
1448152_at	Igf2	5.10	0.0050	Up	16002
1425598_a_at	LOC676654///Lyn	5.07	0.0206	Up	17096///676654
1448201_at	Sfrp2	5.06	0.0035	Up	20319
1455604_at	Fzd5	5.04	0.0236	Up	14367
1452114_s_at	Igfbp5	5.02	0.0055	Up	16011
1425926_a_at	Otx2	5.02	0.0213	Up	18424
1451047_at	Itm2a	5.00	0.0037	Up	16431
1423311_s_at	Tpbg	4.99	0.0082	Up	21983
1455899_x_at	Socs3	4.98	0.0082	Up	12702
1421836_at	Mtap7	4.96	0.0180	Up	17761
1452459_at	Aspm	4.92	0.0080	Up	12316
1450857_a_at	Col1a2	4.92	0.0288	Up	12843
1428481_s_at	Cdca8	4.91	0.0334	Up	52276
1426817_at	Mki67	4.89	0.0186	Up	17345
1458869_at	2900076A13Rik	4.78	0.0259	Up	73002
1449363_at	Atf3	4.78	0.0002	Up	11910
1436221_at	Ildr2	4.74	0.0060	Up	100039795
1437173_at	S1pr3	4.74	0.0115	Up	13610
1418317_at	Lhx2	4.72	0.0086	Up	16870
1416123_at	Ccnd2	4.71	0.0040	Up	12444
1421088_at	Gpc4	4.70	0.0005	Up	14735
1453753_at	Dtl	4.70	0.0286	Up	76843
1434767_at	C79407	4.66	0.0178	Up	217653
1455972_x_at	Hadh	4.51	0.0143	Up	15107
1459894_at	Iqgap2	4.51	0.0151	Up	544963
1452217_at	Ahnak	4.50	0.0059	Up	66395
1417150_at	Slc6a4	4.49	0.0038	Up	15567
1428105_at	Tpx2	4.43	0.0147	Up	72119
1423100_at	Fos	4.39	0.0007	Up	14281
1417073_a_at	Qk	4.39	0.0147	Up	19317
1447488_at		4.26	0.0093	Up	
1418289_at	Nes	4.26	0.0059	Up	18008
1421163_a_at	Nfia	4.23	0.0112	Up	18027
1437244_at	Gas2l3	4.21	0.0056	Up	237436
1420028_s_at	LOC100045677///Mcm3	4.18	0.0065	Up	100045677///17215
1439627_at	Zic1	4.15	0.0237	Up	22771
1437347_at	Ednrb	4.14	0.0278	Up	13618
1437626_at	Zfp36l2	4.14	0.0126	Up	12193
1438303_at	Tgfb2	4.13	0.0316	Up	21808
1440924_at	Kif20b	4.11	0.0375	Up	240641
1436293_x_at	Ildr2	4.11	0.0263	Up	100039795
1435963_at	Sema5b	4.11	0.0067	Up	20357
1436329_at	Egr3	4.11	0.0055	Up	13655
1448734_at	Cp	4.09	0.0167	Up	12870
1417534_at	Itgb5	4.07	0.0004	Up	16419
1431115_at	Tgif2	4.07	0.0031	Up	228839
1448229_s_at	Ccnd2	4.05	0.0247	Up	12444
1426639_a_at	Tcf7l2	4.01	0.0112	Up	21416
1449705_x_at	LOC100045677///Mcm3	3.99	0.0024	Up	100045677///17215
1450781_at	Hmga2	3.98	0.0068	Up	15364
1448606_at	Lpar1	3.96	0.0042	Up	14745
1449289_a_at	B2m	3.95	0.0029	Up	12010
1428142_at	Etv5	3.92	0.0093	Up	104156
1425811_a_at	Csrp1	3.92	0.0187	Up	13007
1416309_at	Nusap1	3.91	0.0222	Up	108907
1421317_x_at	Myb	3.91	0.0273	Up	17863
1423586_at	Axl	3.89	0.0002	Up	26362
1417419_at	Ccnd1	3.88	0.0228	Up	12443
1422929_s_at	Atoh7	3.87	0.0095	Up	53404
1450379_at	Msn	3.83	0.0013	Up	17698
1448363_at	Yap1	3.82	0.0007	Up	22601
1455990_at	Kif23	3.82	0.0069	Up	71819
1428786_at	Nckap1l	3.80	0.0034	Up	105855
1425457_a_at	Grb10	3.79	0.0182	Up	14783
1450843_a_at	Serpinh1	3.78	0.0023	Up	12406
1415945_at	Mcm5	3.77	0.0066	Up	17218
1417911_at	Ccna2	3.77	0.0354	Up	12428
1419943_s_at	Ccnb1	3.77	0.0110	Up	268697
1449577_x_at	Tpm2	3.76	0.0048	Up	22004
1423852_at	Shisa2	3.76	0.0050	Up	219134
1460291_at	Cdk6	3.74	0.0015	Up	12571
1424603_at	Sumf1	3.72	0.0265	Up	58911
1448519_at	Tead2	3.72	0.0072	Up	21677
1417506_at	Gmnn	3.70	0.0248	Up	57441
1416340_a_at	Man2b1	3.69	0.0020	Up	17159
1419944_at	Ccnb1	3.69	0.0190	Up	268697
1426341_at	Slc1a3	3.65	0.0013	Up	20512
1417395_at	Klf4	3.65	0.0030	Up	16600
1429189_at	Arsb	3.65	0.0062	Up	11881
1436708_x_at	Mcm4	3.64	0.0223	Up	17217
1442340_x_at	Cyr61	3.62	0.0172	Up	16007
1418912_at	Plxdc2	3.59	0.0032	Up	67448
1417494_a_at	Cp	3.59	0.0260	Up	12870
1454834_at	Nfib	3.58	0.0047	Up	18028
1452954_at	Ube2c	3.58	0.0329	Up	68612
1420904_at	Il17ra	3.56	0.0026	Up	16172
1434079_s_at	Mcm2	3.55	0.0083	Up	17216
1455287_at	Cdk6	3.54	0.0154	Up	12571
1424099_at	Gpx8	3.53	0.0044	Up	69590
1448883_at	Lgmn	3.49	0.0125	Up	19141
1424629_at	Brca1	3.46	0.0227	Up	12189
1448627_s_at	Pbk	3.44	0.0083	Up	52033
1417420_at	Ccnd1	3.43	0.0220	Up	12443
1447839_x_at	Adm	3.42	0.0179	Up	11535
1456733_x_at	Serpinh1	3.42	0.0075	Up	12406
1430164_a_at	Grb10	3.42	0.0122	Up	14783
1435176_a_at	Id2	3.40	0.0062	Up	15902
1456772_at	Ncf1	3.38	0.0025	Up	17969
1443047_at		3.38	0.0001	Up	
1436847_s_at	Cdca8	3.35	0.0299	Up	52276
1459713_s_at	Ano1	3.35	0.0188	Up	101772
1416006_at	Mdk	3.32	0.0078	Up	17242
1442280_at	D2Ertd750e	3.30	0.0093	Up	51944
1418049_at	Ltbp3	3.28	0.0175	Up	16998
1438588_at	Plagl1	3.27	0.0301	Up	22634
1427276_at	Smc4	3.24	0.0269	Up	70099
1416846_a_at	Pdzrn3	3.22	0.0341	Up	55983
1427275_at	Smc4	3.22	0.0204	Up	70099
1419647_a_at	Ier3	3.22	0.0013	Up	15937
1415810_at	Uhrf1	3.22	0.0051	Up	18140
1423250_a_at	Tgfb2	3.21	0.0232	Up	21808
1434945_at	Lpcat2	3.21	0.0223	Up	270084
1450082_s_at	Etv5	3.20	0.0007	Up	104156
1417450_a_at	Tacc3	3.20	0.0318	Up	21335
1455154_at	Gli3	3.19	0.0149	Up	14634
1437687_x_at	Fkbp9	3.19	0.0013	Up	27055
1426600_at	Slc2a1	3.19	0.0159	Up	20525
1448698_at	Ccnd1	3.13	0.0332	Up	12443
1448620_at	Fcgr3	3.13	0.0068	Up	14131
1423608_at	Itm2a	3.12	0.0292	Up	16431
1416757_at	Zwilch	3.09	0.0066	Up	68014
1417533_a_at	Itgb5	3.09	0.0087	Up	16419
1434936_at	Hirip3	3.09	0.0271	Up	233876
1452540_a_at	Hist1h2bc///Hist1h2be///Hist1h2bl///Hist1h2bm///Hist1h2bp///LOC100046213///LOC665622///RP23-38E20.1	3.08	0.0272	Up	100046213///319179///319185///319186///319188///665596///665622///68024
1417821_at	D17H6S56E-5	3.07	0.0195	Up	110956
1419700_a_at	Prom1	3.07	0.0023	Up	19126
1437418_at	100041799	3.06	0.0022	Up	100041799
1417133_at	Pmp22	3.06	0.0289	Up	18858
1422432_at	Dbi	3.06	0.0042	Up	13167
1450481_at	Mybl1	3.05	0.0335	Up	17864
1460220_a_at	Csf1	3.05	0.0013	Up	12977
1416440_at	Cd164	3.03	0.0022	Up	53599
1423593_a_at	Csf1r	3.02	0.0035	Up	12978
1450920_at	Ccnb2	3.02	0.0163	Up	12442
1417495_x_at	Cp	3.02	0.0131	Up	12870
1424991_s_at	Tyms///Tyms-ps	3.00	0.0181	Up	22171///22172
1448148_at	Grn	3.00	0.0070	Up	14824
1436514_at	Gpc4	3.00	0.0172	Up	14735
1429171_a_at	Ncapg	2.96	0.0178	Up	54392
1426246_at	Pros1	2.94	0.0004	Up	19128
1423775_s_at	Prc1	2.93	0.0300	Up	233406
1418633_at	Notch1	2.93	0.0120	Up	18128
1454714_x_at	EG665516///EG666036///EG668771///Phgdh	2.92	0.0090	Up	236539///665516///666036///668771
1437478_s_at	Efhd2	2.92	0.0031	Up	27984
1416368_at	Gsta4	2.92	0.0003	Up	14860
1450686_at	Pon2	2.91	0.0118	Up	330260
1450020_at	Cx3cr1	2.90	0.0203	Up	13051
1452040_a_at	Cdca3	2.90	0.0375	Up	14793
1418340_at	Fcer1g	2.87	0.0006	Up	14127
1422445_at	Itga6	2.87	0.0006	Up	16403
1420643_at	Lfng	2.86	0.0033	Up	16848
1448314_at	Cdc2a	2.86	0.0183	Up	12534
1444257_at	Prr11	2.84	0.0147	Up	270906
1428227_at	Rest	2.84	0.0252	Up	19712
1423298_at	Add3	2.81	0.0051	Up	27360
1451080_at	Usp1	2.81	0.0232	Up	230484
1429190_at	Arsb	2.80	0.0032	Up	11881
1433492_at	Epb4.1l2	2.79	0.0019	Up	13822
1448232_x_at	100042266///EG434428///EG636070///LOC100044416///LOC100045728///Tuba1a///Tuba1b///Tuba1c	2.78	0.0019	Up	100042266///100044416///100045728///22142///22143///22146///434428///636070
1417947_at	Pcna	2.77	0.0086	Up	18538
1422444_at	Itga6	2.76	0.0054	Up	16403
1420820_at	2900073G15Rik	2.75	0.0247	Up	67268
1455393_at	Cp	2.75	0.0072	Up	12870
1433490_s_at	Epb4.1l2	2.75	0.0027	Up	13822
1433857_at	Fat1	2.74	0.0126	Up	14107
1417985_at	Nrarp	2.74	0.0134	Up	67122
1452035_at	Col4a1	2.73	0.0024	Up	12826
1452404_at	Phactr2	2.73	0.0040	Up	215789
1422706_at	Pmepa1	2.73	0.0139	Up	65112
1434474_at	Abca5	2.73	0.0042	Up	217265
1416630_at	Id3	2.72	0.0021	Up	15903
1417065_at	Egr1	2.70	0.0052	Up	13653
1442728_at		2.70	0.0124	Up	
1422612_at	Hk2	2.69	0.0005	Up	15277
1424604_s_at	Sumf1	2.68	0.0215	Up	58911
1425271_at	Psmc3ip	2.68	0.0137	Up	19183
1450533_a_at	Plagl1	2.67	0.0280	Up	22634
1456567_x_at	Grn	2.67	0.0009	Up	14824
1448474_at	Nek7	2.65	0.0055	Up	59125
1435578_s_at	Dab1	2.64	0.0240	Up	13131
1416724_x_at	Tcf4	2.64	0.0110	Up	21413
1449888_at	Epas1///LOC100048537	2.64	0.0166	Up	100048537///13819
1417878_at	E2f1	2.64	0.0327	Up	13555
1417483_at	Nfkbiz	2.63	0.0185	Up	80859
1452881_at	Gins2	2.62	0.0103	Up	272551
1449140_at	Nudcd2	2.62	0.0150	Up	52653
1444800_at		2.61	0.0099	Up	
1416251_at	Mcm6	2.61	0.0322	Up	17219
1423414_at	Ptgs1	2.59	0.0093	Up	19224
1420171_s_at	Myh9	2.58	0.0015	Up	17886
1460184_at	Hadh	2.57	0.0092	Up	15107
1425458_a_at	Grb10	2.56	0.0193	Up	14783
1417822_at	D17H6S56E-5	2.56	0.0079	Up	110956
1418634_at	Notch1	2.53	0.0080	Up	18128
1425314_at	Gpr98	2.53	0.0256	Up	110789
1459740_s_at	Ucp2	2.52	0.0141	Up	22228
1427762_x_at	Hist1h2bp	2.52	0.0278	Up	319188
1423596_at	Nek6	2.51	0.0283	Up	59126
1422938_at	Bcl2	2.51	0.0110	Up	12043
1448272_at	Btg2	2.51	0.0009	Up	12227
1416214_at	Mcm4	2.49	0.0136	Up	17217
1434069_at	Prex1	2.49	0.0081	Up	277360
1423675_at	Usp1	2.49	0.0195	Up	230484
1448259_at	Fstl1	2.47	0.0077	Up	14314
1416433_at	Rpa2	2.47	0.0102	Up	19891
1422016_a_at	Cenph	2.46	0.0347	Up	26886
1432604_at	Rbl1	2.46	0.0293	Up	19650
1422695_at	Ttyh1	2.45	0.0131	Up	57776
1438629_x_at	Grn	2.45	0.0006	Up	14824
1424089_a_at	Tcf4	2.45	0.0056	Up	21413
1416498_at	Ppic	2.44	0.0289	Up	19038
1420824_at	Sema4d	2.43	0.0116	Up	20354
1416431_at	Tubb6	2.41	0.0185	Up	67951
1454830_at	Fbn2	2.40	0.0065	Up	14119
1423660_at	Ctdsp2///ENSMUSG00000040540	2.40	0.0178	Up	100043719///52468
1420172_at		2.39	0.0069	Up	
1444785_at		2.39	0.0120	Up	
1422831_at	Fbn2	2.39	0.0065	Up	14119
1445597_s_at	Pla2g16	2.39	0.0075	Up	225845
1418690_at	Ptprz1	2.39	0.0015	Up	19283
1433991_x_at	Dbi	2.38	0.0241	Up	13167
1421814_at	Msn	2.38	0.0088	Up	17698
1429556_at	2610024B07Rik	2.38	0.0154	Up	269987
1446172_at		2.37	0.0091	Up	
1418099_at	Tnfrsf1b	2.36	0.0222	Up	21938
1420959_at	Asph	2.36	0.0280	Up	65973
1426936_at	629242///BC005512///F630007L15Rik	2.36	0.0322	Up	192885///629242///641366
1438093_x_at	Dbi	2.35	0.0242	Up	13167
1426653_at	LOC100045677///Mcm3	2.34	0.0146	Up	100045677///17215
1453007_at	3110082I17Rik	2.34	0.0141	Up	73212
1416653_at	LOC100047484///Stxbp3a	2.33	0.0028	Up	100047484///20912
1420774_a_at	4930583H14Rik	2.33	0.0098	Up	67749
1421867_at	Nr3c1	2.32	0.0021	Up	14815
1426195_a_at	Cst3	2.32	0.0009	Up	13010
1428029_a_at	H2afv	2.32	0.0121	Up	77605
1442058_s_at	Psmc3ip	2.32	0.0022	Up	19183
1429270_a_at	Syce2	2.31	0.0096	Up	71846
1448139_at	Mlc1	2.30	0.0218	Up	170790
1416049_at	Gldc	2.29	0.0171	Up	104174
1447878_s_at	Fgfrl1///LOC100046239	2.28	0.0098	Up	100046239///116701
1442215_at	Smo	2.28	0.0124	Up	319757
1433491_at	Epb4.1l2	2.28	0.0027	Up	13822
1422537_a_at	Id2	2.27	0.0003	Up	15902
1439453_x_at	Rnaseh2c	2.27	0.0284	Up	68209
1434768_at	Tpp1	2.27	0.0060	Up	12751
1456245_x_at	Vamp3	2.27	0.0352	Up	22319
1456439_x_at	Mical1	2.27	0.0059	Up	171580
1453183_at	1110034A24Rik	2.26	0.0056	Up	109065
1452928_at	Abi3	2.26	0.0098	Up	66610
1442003_at	Diap2	2.25	0.0028	Up	54004
1428670_at	LOC72520	2.25	0.0136	Up	72520
1455976_x_at	Dbi	2.25	0.0216	Up	13167
1438852_x_at	Mcm6	2.23	0.0242	Up	17219
1434150_a_at	Higd1c///Mettl7a1///Mettl7a2	2.22	0.0070	Up	380975///393082///70152
1439436_x_at	Incenp	2.22	0.0275	Up	16319
1455956_x_at	Ccnd2	2.22	0.0156	Up	12444
1460247_a_at	Skp2	2.21	0.0335	Up	27401
1416986_a_at	Sirpa	2.21	0.0077	Up	19261
1437894_at	Prox1	2.20	0.0074	Up	19130
1417472_at	Myh9	2.20	0.0054	Up	17886
1422881_s_at	Sypl	2.20	0.0046	Up	19027
1423674_at	Usp1	2.19	0.0341	Up	230484
1425565_at	Rest	2.19	0.0031	Up	19712
1437865_at	Spata13	2.19	0.0038	Up	219140
1437708_x_at	Vamp3	2.19	0.0093	Up	22319
1439060_s_at	Wipi1	2.18	0.0017	Up	52639
1416221_at	Fstl1	2.16	0.0008	Up	14314
1416250_at	Btg2	2.16	0.0304	Up	12227
1437623_x_at	Xrcc3	2.16	0.0082	Up	74335
1460559_at	Kank2	2.16	0.0361	Up	235041
1428853_at	Ptch1	2.16	0.0125	Up	19206
1433645_at	Slc44a1	2.15	0.0013	Up	100434
1452671_s_at	Lman1	2.15	0.0210	Up	70361
1442014_at		2.15	0.0093	Up	
1450641_at	Vim	2.14	0.0275	Up	22352
1454777_at	Slco2b1	2.13	0.0158	Up	101488
1428976_at	Tmpo	2.13	0.0374	Up	21917
1456133_x_at	Itgb5	2.13	0.0020	Up	16419
1444443_at		2.13	0.0004	Up	
1460344_at	2310033F14Rik	2.13	0.0044	Up	69555
1420380_at	Ccl2	2.12	0.0057	Up	20296
1416985_at	Sirpa	2.12	0.0046	Up	19261
1423424_at	Zic3	2.12	0.0321	Up	22773
1423493_a_at	Nfix	2.11	0.0328	Up	18032
1426412_at	Neurod1	2.11	0.0144	Up	18012
1422286_a_at	Tgif1	2.11	0.0200	Up	21815
1418534_at	Fzd2	2.11	0.0030	Up	57265
1416122_at	Ccnd2	2.10	0.0002	Up	12444
1416076_at	Ccnb1///EG434175///EG667005	2.10	0.0272	Up	268697///434175///667005
1439562_at	F730047E07Rik	2.09	0.0228	Up	212377
1415834_at	Dusp6	2.09	0.0051	Up	67603
1447448_s_at	Klf6	2.09	0.0134	Up	23849
1448118_a_at	Ctsd	2.09	0.0129	Up	13033
1447852_x_at	Rilpl1	2.08	0.0025	Up	75695
1429295_s_at	Trip13	2.08	0.0320	Up	69716
1419951_at	Lman1	2.08	0.0322	Up	70361
1442134_at	Prr11	2.08	0.0301	Up	270906
1416992_at	LOC100046464///Mfng	2.08	0.0013	Up	100046464///17305
1434149_at	Tcf4	2.08	0.0328	Up	21413
1433916_at	Vamp3	2.08	0.0244	Up	22319
1415691_at	Dlg1///LOC100047603	2.07	0.0173	Up	100047603///13383
1420970_at	Adcy7	2.07	0.0023	Up	11513
1449127_at	Selplg	2.07	0.0195	Up	20345
1435657_at	Ston2	2.07	0.0162	Up	108800
1434503_s_at	Lamp2	2.05	0.0169	Up	16784
1423871_at	Tmem63a	2.04	0.0002	Up	208795
1417870_x_at	Ctsz	2.04	0.0085	Up	64138
1443231_at		2.03	0.0323	Up	
1425166_at	Rbl1	2.03	0.0252	Up	19650
1417709_at	Cyp46a1	2.02	0.0017	Up	13116
1424265_at	Npl	2.02	0.0064	Up	74091
1424299_at	Oma1	2.02	0.0198	Up	67013
1423434_at	Tead1	2.02	0.0279	Up	21676
1448777_at	Mcm2	2.02	0.0375	Up	17216
1453314_x_at	2610039C10Rik	2.02	0.0150	Up	66578
1417470_at	Apobec3	2.01	0.0301	Up	80287
1429894_a_at	Mtap7	2.01	0.0043	Up	17761
1428094_at	Lamp2	2.01	0.0027	Up	16784
1435526_at	Tor1aip2	2.00	0.0095	Up	240832
1440270_at	Fgf12	-2.02	0.0099	Down	14167
1453856_at	Zbtb46	-2.03	0.0149	Down	72147
1441949_x_at	Slc39a6	-2.03	0.0136	Down	106957
1459903_at	Sema7a	-2.03	0.0216	Down	20361
1429464_at	Prkaa2	-2.04	0.0028	Down	108079
1437982_x_at	Cox15	-2.05	0.0340	Down	226139
1458464_at	Hecw2	-2.05	0.0251	Down	329152
1419717_at	Sema3e	-2.06	0.0053	Down	20349
1450151_at	Zfp316	-2.06	0.0189	Down	54201
1437401_at	Igf1	-2.06	0.0083	Down	16000
1435064_a_at	Tmem27	-2.07	0.0097	Down	57394
1423328_at	Gdap1	-2.09	0.0084	Down	14545
1438664_at	Prkar2b	-2.10	0.0329	Down	19088
1418288_at	Lpin1	-2.11	0.0343	Down	14245
1426446_at	6430548M08Rik	-2.11	0.0262	Down	234797
1435974_at	Arhgef9	-2.13	0.0215	Down	236915
1434027_at	Rcan3	-2.13	0.0225	Down	53902
1425158_at	Tbx20	-2.15	0.0015	Down	57246
1455194_at	Mapk8ip2	-2.16	0.0182	Down	60597
1433601_at	Adra2a	-2.18	0.0341	Down	11551
1455633_at	Zfp647	-2.18	0.0182	Down	239546
1420955_at	Vsnl1	-2.19	0.0225	Down	26950
1429668_at	Pou4f1	-2.20	0.0005	Down	18996
1436937_at	Rbms3	-2.20	0.0318	Down	207181
1427673_a_at	Sema3e	-2.20	0.0034	Down	20349
1438697_at	Tmem132c	-2.22	0.0244	Down	208213
1424482_at	Arhgef7	-2.22	0.0047	Down	54126
1420679_a_at	Aig1	-2.23	0.0077	Down	66253
1457793_a_at	Whsc1l1	-2.26	0.0347	Down	234135
1419458_at	Rgnef	-2.27	0.0302	Down	110596
1436014_a_at	Rusc1	-2.28	0.0071	Down	72296
1439852_at		-2.31	0.0237	Down	
1429696_at	Gpr123	-2.33	0.0153	Down	52389
1444923_at		-2.35	0.0203	Down	
1418847_at	Arg2	-2.37	0.0065	Down	11847
1420448_at	Rhox2a	-2.39	0.0365	Down	75199
1455358_at	A2bp1	-2.44	0.0000	Down	268859
1417679_at	Gfi1	-2.47	0.0028	Down	14581
1447788_s_at	Tspyl3	-2.51	0.0243	Down	241732
1442226_at	Sema3e	-2.52	0.0358	Down	20349
1458623_at		-2.53	0.0159	Down	
1428568_at	B230217C12Rik	-2.63	0.0015	Down	68127
1416286_at	Rgs4	-2.68	0.0023	Down	19736
1434413_at	Igf1	-2.77	0.0009	Down	16000
1429273_at	Bmper	-2.83	0.0025	Down	73230
1447552_s_at		-3.01	0.0137	Down	
1452444_at	Napb	-3.05	0.0172	Down	17957

Genes that were enriched by 2-fold or more with corrected p-value <=0.05 in either the ipsilateral or contralateral RGC population were selected for this list.

### Genes selectively expressed in contralateral RGCs

**Figure 4.**
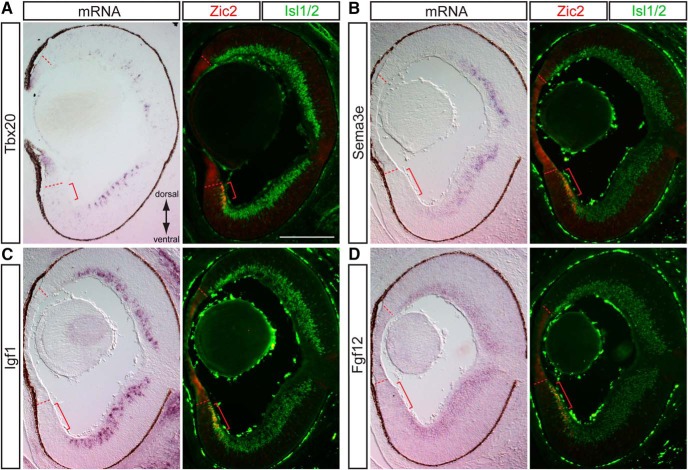
Genes enriched in the contralateral RGC population. ISH analysis at E15.5 shows complementary expression of Tbx20 (***A***), Sema3e (***B***), and Igf1 mRNA (contralateral RGCs) with Zic2 (ipsilateral RGCs). Fgf12 is highly expressed in Zic2^–^ RGCs, with only trace levels of expression in Zic2^+^ cells. All candidate genes are expressed in Islet1/2^+^ (differentiated) RGCs. Scale bars, 250 μm.

At present, very few specific markers of contralateral RGCs have been identified, and no gene has been shown to specify contralateral identity throughout the retina. To confirm that select candidate genes are indeed specifically expressed in contralateral RGCs, we analyzed the mRNA expression pattern of the gene of interest at E15.5 (peak of Zic2 expression) by ISH followed by co-immunostaining with ipsilateral RGC marker Zic2 and pan-RGC marker Islet1/2 within the same sections. We selected these seven genes based on the magnitude of their fold change (Napb, Bmper, and Igf1 were the most highly enriched in contralateral RGCs) as well as their potential function during development as predicted by their known functional classification roles in other systems. Of the seven new contralateral genes tested, the following genes showed exclusive expression in contralateral RGCs (Islet1/2^+^Zic2^–^ cells): transcription factor Tbx20, cell surface/secreted protein Sema3e, and growth factor Igf1 (Fig. 4*A–C*). Fgf12 is also enriched in RGCs outside of the Zic2^+^ zone and shows only weak expression in VT RGCs (Fig. 4*D*). Interestingly, Fgf12 and Igf1 expression is weaker in the most peripheral RGCs of dorsotemporal (DT) retina, suggesting that the youngest DT RGCs (Fig. 4*C*, *D*, adjacent to dashed red line) have not yet accumulated as much of these transcripts as the more central RGCs. Tbx20 and Sema3e are even more centrally expressed and appear to have more mosaic expression. None of these genes have been studied in the context of RGC development or contralateral RGC function. Together, Igf1, Fgf12, Tbx20, and Sema3e represent four new contralateral RGC markers during the peak period of axon outgrowth, when ipsilateral and contralateral RGC axons diverge.

### Genes selectively expressed in ipsilateral RGCs

To validate the expression pattern of genes enriched in ipsilateral RGCs in the microarray, we again used ISH analysis on coronal sections of E15.5 retina, co-immunostaining for RGC markers. Here, we selected transcription factors that participate in CNS development as discussed above, as well as cell cycle regulators, developmental signaling molecules, axon guidance molecules, and other secreted or membrane-expressed genes that may play a role in differentiated RGC function. Of the 23 new ipsilateral genes tested, many were expressed in proliferative zones within the retina, as expected. However, three genes, Sox2, Math5, and Igfbp5, were additionally expressed in differentiated ipsilateral RGCs. We also detected four other genes, Lhx2, Zic1, Gja1, and Ccnd2, that were expressed in ipsilateral RGCs either at a weaker level or within a more limited developmental window (data not shown).

**Figure 5.**
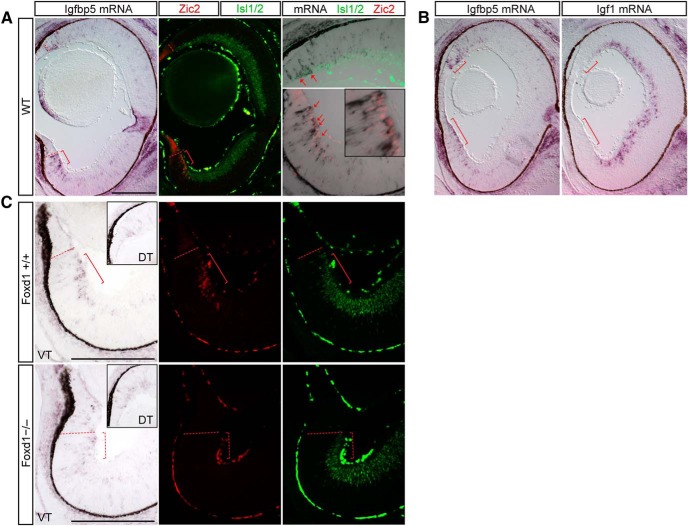
Igfbp5 is expressed in the VT RGC zone in Zic2^+^ cells. ISH analysis at E15.5. ***A***, Igfbp5 mRNA is expressed in a subset of Zic2^+^ RGCs in VT retina (red arrows) and a few Zic2^–^ RGCs in dorsal retina. Red brackets mark the ipsilateral RGC domain. ***B***, Igfbp5 (red brackets) and Igf1 are expressed in a complementary pattern throughout the retina. ***C***, Igfbp5 expression is concomitantly reduced in the Foxd1 KO mutant compared with WT littermates; this reduction correlates with the similar reduction in Zic2 expression in the VT retina of the Foxd1 KO mutant. Red brackets mark the expected ipsilateral RGC domain. Scale bars, 250 μm.

The most striking expression pattern of this group of validated genes is that of insulin-like growth factor binding protein 5 (Igfbp5). Igfbp5 is expressed in RGCs of the Zic2^+^ RGC zone throughout the VT retina (Fig 5*A*). Closer examination reveals that Igfbp5 is most strongly expressed in Zic2^+^Islet1/2^+^ RGCs of this region, but not all Zic2^+^ RGCs express Igfbp5, and some Zic2^–^ cells also express lower levels of Igfbp5 and are located at the junction between the RGC and neuroblastic layers (Fig. 5*A*, high power). Igfbp5 is also expressed in the most peripheral contralaterally projecting RGCs (Zic2^–^Islet1/2^+^) of DT retina, although these cells are much fewer than the VT RGCs that express Igfbp5. Interestingly, Igfbp5^+^ cells correspond to the most peripheral DT RGCs that lack Igf1 expression (Fig. 5*B*). Thus, Igfbp5 and Igf1 have a complementary pattern of expression in E15.5 retina, suggesting that these two components of the Igf signaling pathway have complementary or antagonistic functions at this age.

To determine whether Igfbp5 is truly related to ipsilateral RGC identity, we studied the expression of Igfbp5 in the Foxd1 knockout, in which the patterning of the region containing ipsilateral RGCs is disrupted with concomitant loss of Zic2-expressing RGCs. Indeed, Igfbp5 coexpression within RGCs is reduced within the VT retina (Fig. 5*C*), suggesting that Igfbp5 is not merely expressed in the peripheral-most RGCs but rather is uniquely upregulated within ipsilateral RGCs.

### Ipsilateral RGCs express early developmental markers

**Figure 6.**
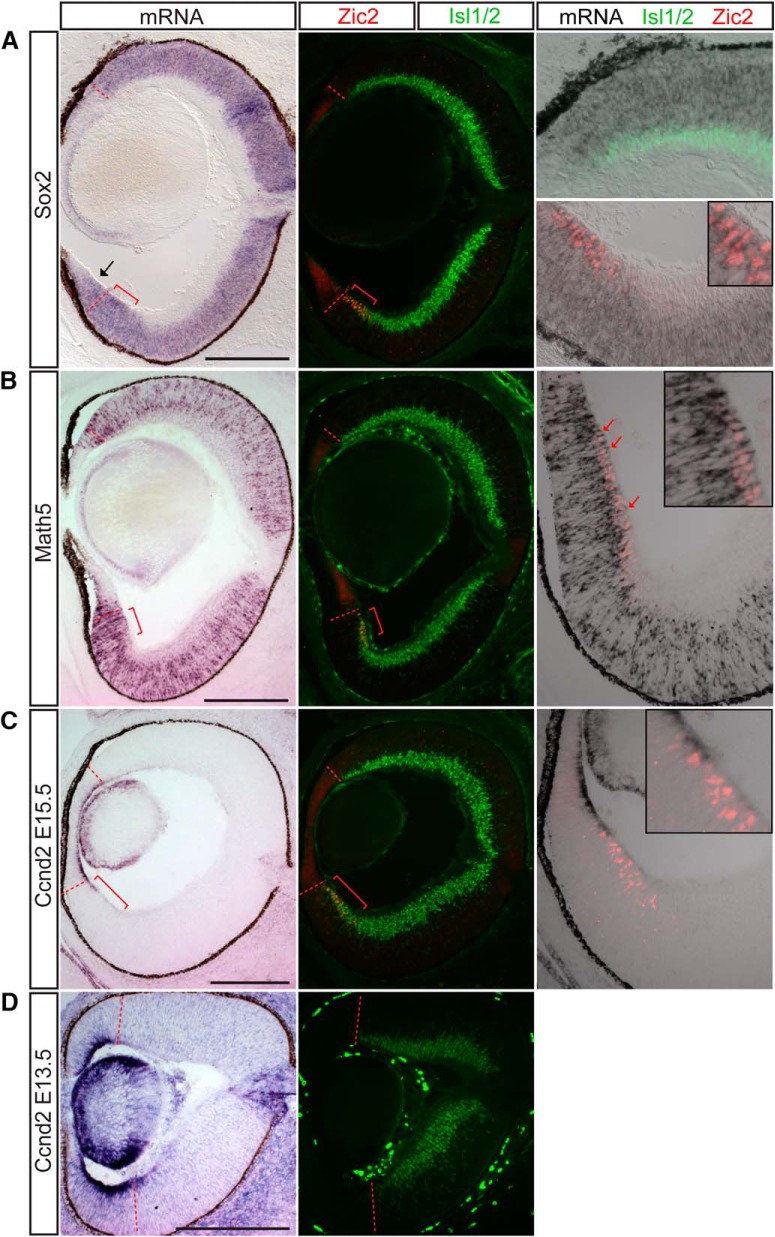
Sox2, Math5, and cyclin D2 are enriched in the ipsilateral RGC population in addition to progenitor cells throughout the retina. ISH analysis at E15.5. ***A***, Sox2 mRNA is expressed in the neuroblastic layer and Zic2^+^ RGCs but not Zic2^–^/Islet1/2^+^ (differentiated) RGCs extra-VT retina. ***B***, Similarly, Math5 mRNA is expressed in Zic2^+^ RGCs located at the periphery of the VT retina in the RGC layer as well as in RGC precursors in the neuroblastic layer (red arrows). ***C***, Cyclin D2 mRNA is expressed in the basal process of cells within the peripheral margin of the retina, particularly in the ventral retina, and also at low levels in the Zic2^+^ RGC zone at E15.5. ***D***, The asymmetric expression of cyclin D2, with higher levels in ventral retina, is more pronounced at E13.5. Red brackets mark the Zic2^+^ ipsilateral RGC domain. Scale bars, 250 μm.

A large group of genes enriched in ipsilateral RGCs in the microarray were transcription factors known to be important in the development of retinal progenitor cells or RGC precursors. Two possible explanations for this finding include: (1) the ipsilateral RGC sample is disproportionately contaminated with RNA from proliferating cells, or (2) these genes are indeed enriched in ipsilateral RGCs. In support of the latter scenario, two ipsilateral RGC-enriched microarray candidates, Sox2 and Math, show clear expression in both progenitor cells and differentiated RGCs (Fig. 6*A*, *B*). Sox2 is expressed in neuronal progenitors during CNS development, including the retina, but is downregulated during the final division as the progenitor becomes postmitotic (Pevny and Nicolis, 2010). Indeed, we observed homogeneous Sox2 expression within the proliferative regions of retina, i.e., the neuroblastic layer progenitors, ciliary body progenitors (Fig. 6*A*), and optic nerve head glial progenitors (not shown). Remarkably, Sox2 is also expressed homogeneously and at an overall stronger level within the Zic2^+^ VT RGC zone and colocalizes with Zic2 and Islet1/2 expression. Sox2 is completely absent from DT RGCs, indicated by Islet1/2 expression, including the most peripheral cells. Thus, Sox2 mRNA expression is maintained in postmitotic Zic2^+^ RGCs and, similar to SERT, is a distinct marker of this RGC subset.

The transcription factor Math5 is expressed in retinal progenitors after they acquire competence to generate RGCs (Wang et al., 2001; Brzezinski et al., 2012) and thus marks the first neurogenic competence state of retinal progenitors (Yang et al., 2003). Similar to Sox2, Math5 mRNA expression extends into the Zic2^+^ RGC zone. However, unlike the homogeneous expression of Sox2, Math5 colocalizes with Zic2 expression predominantly in peripheral RGCs (Fig. 6*B*, red arrows indicate Zic2^+^ nucleus in red with surrounding Math5 mRNA in black). This colocalization is not as pronounced in RGCs in DT retina.

Sox2 and Math5 expression patterns indicate that ipsilateral RGCs retain the expression of genes initially expressed before differentiation. This may be due to an upstream regulatory program that differs between ipsilateral and contralateral RGCs, turning off the expression of progenitor genes within contralateral RGCs but allowing for their continued expression in ipsilateral RGCs. These results raise the intriguing question of why Zic2^+^ ipsilaterally projecting RGCs maintain the expression of such genes. Interestingly, we detected a number of cell cycle regulators enriched in ipsilateral RGCs in our microarray screen. In particular, the G1 cyclin D2 stood out as an interesting candidate, as its function has not been previously examined in the retina due to the lack of a gross retinal phenotype in cyclin D2 null mice (Sicinski et al., 1996). In contrast, mice without cyclin D1, which is the predominant G1 cyclin during retinal development, have severely hypocellular retinas (Fantl et al., 1995; Sicinski et al., 1995; Dyer and Cepko, 2001; Geng et al., 2001; Das et al., 2009).

In our ISH studies, cyclin D2 (Ccnd2) is expressed asymmetrically in the proliferating cells peripheral to the RGC zone at E15.5, with a higher level and broader zone of expression in ventral retina compared with dorsal retina (Fig. 6*C*; see also Trimarchi et al., 2009). Moreover, lower levels of cyclin D2 mRNA expression extend into the Zic2^+^ RGC zone but can also be detected at a lower level within the entire span of the Zic2^+^ RGC zone (Fig. 6*C*, inset). The asymmetry in expression and extension of the cyclin D2 domain into the Zic2^+^ RGC zone are even more pronounced at E13.5 (Fig. 6*D*). Because of the localization of Ccnd2 mRNA to the basal process of these cells, which has been previously described in the cortex (Tsunekawa et al., 2012), we are unable to determine whether Cyclin D2 mRNA and Zic2 protein expression colocalize in the same cells.

**Figure 7.**
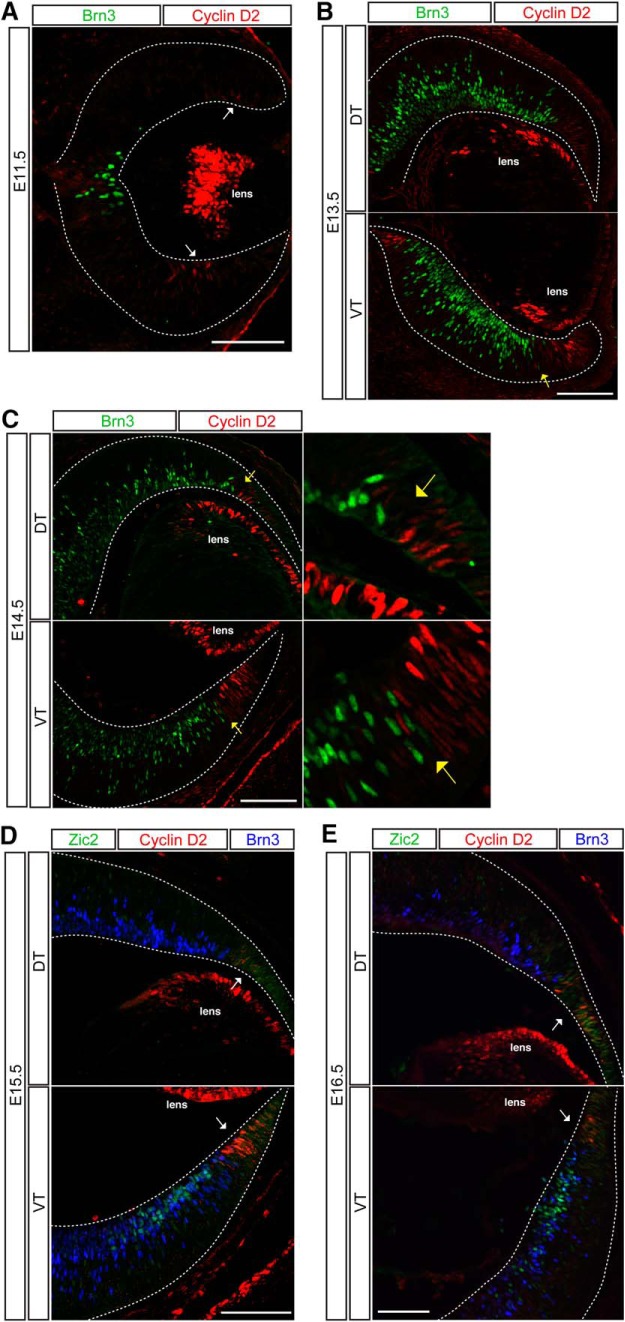
Cyclin D2 is enriched in the ventral peripheral retina during the temporal window of ipsilateral RGC genesis. IHC in coronal cryosections of E11.5 (***A***), E12.5 (***B***), E14.5 (***C***), E15.5 (***D***), and E16.5 (***E***) retina. Cyclin D2 is expressed in the lens and the retinal marginal zone bordering RGCs (labeled with Brn3). Cyclin D2 is highly expressed in the ventral periphery compared with the dorsal periphery within neural retina (white arrows). The cyclin D2 and Brn3 domains are separated by a large gap at E11.5 (***A***). At E13.5 (***B***) and E14.5 (***C***), cyclin D2^+^ cells intermingle with Brn3^+^ RGCs only in ventral retina (yellow arrows point to boundary) and have sharp boundaries in dorsal retina. At E16.5 (***E***), cyclin D2 expression is reduced in the retinal periphery and is no longer asymmetric in dorsal and ventral retinal (white arrows). Scale bars, 100 μm.

To determine cyclin D2 protein localization throughout development, retinal sections from E11.5, 13.5, 14.5, 15.5, and E16.5 embryos were immunostained with antibodies against cyclin D2, Zic2 (ipsilateral RGCs), and Brn3 (all RGCs). Consistent with the ISH results, IHC analysis showed that cyclin D2 is expressed within the retinal periphery and the optic nerve head, and its expression is substantially higher in ventral than in dorsal periphery until E15.5 (Fig. 7*A–D*). At E16.5, this asymmetry in the ventro-dorsal expression of cyclin D2 decreases, as fewer cells now express cyclin D2 in ventral retina (Fig. 7*E*). In contrast to the ISH results, immunostaining revealed that cyclin D2 protein is nuclear rather than at the basal surface, with cells expressing the highest levels of cyclin D2 positioned more basally. These distinct cyclin D2 mRNA and protein expression patterns have been similarly reported in cortex (Tsunekawa and Osumi, 2012). Cyclin D2^+^ cells that showed positive staining for Zic2 and Islet1/2 were very rare, indicating that cyclin D2 is expressed predominantly in cells that are situated in the periphery of VT retina, adjacent to the zone of Zic2-expressing cells, and do not yet express markers of differentiated RGCs such as Brn3 or Islet1/2. The finding that cyclin D2 is enriched in the ipsilateral RGC population in our microarray analysis and subsequent confirmation by ISH may reflect the perdurance of mRNA expression in differentiated ipsilateral RGCs. The downregulation of cyclin D2 protein expression in differentiated RGCs that express Zic2 and Islet1/2 may be additionally explained by posttranscriptional regulatory mechanisms.

In contrast to cyclin D2, cyclin D1 expression is homogeneous throughout proliferating regions in the retina (data not shown). Cyclin D2^+^ cells are also positive for cyclin D1. The broad expression of cyclin D1 is consistent with its role as the major D cyclin in retinal development required for general retinal histogenesis (Sicinski et al., 1995; Fantl et al., 1995). The additional expression of cyclin D2 in a specific subset of cells within the retina, however, suggests that it conveys an added layer of regulation on the cell cycle kinetics of these cells.

## Discussion

The increasing availability of transcriptomic technologies within the last decade has facilitated high-throughput identification of gene expression profiles that define distinct cell types (Trimarchi et al., 2007; Okaty et al., 2011; Goetz and Trimarchi, 2012; Mizeracka et al., 2013; Islam et al., 2014; Sümbül et al., 2014; Macosko et al., 2015; Molyneaux et al., 2015; Mullally et al., 2016; Shima et al., 2016). Here we have applied DNA microarray analysis to RGCs projecting ipsilaterally and contralaterally during embryonic development. By combining retrograde labeling and cell sorting, we were able to overcome the challenge of isolating a disproportionately sparse neuronal subtype—the ipsilateral RGC population (3–5% of total RGCs in mouse retina)—and then compared the molecular identity of this population with their contralateral countertypes. These gene-profiling experiments confirmed genes known to be unique to ipsilateral or contralateral RGCs and allowed us to uncover nearly 300 genes that are differentially expressed in these two populations. A number of these genes were subsequently confirmed by endogenous expression in developing retina, thus having high biological relevance. In particular, the new RGC markers expressed solely in contralateral RGCs that we identified fill a significant void, as most genes that have been previously described as important for contralateral RGC function are not exclusively expressed in these cell types but rather are also expressed in ipsilateral RGCs. Among the differentially expressed genes we identified, we have found at least one pair, Igf1 and Igfbp5, that show complementary expression within contralateral and ipsilateral RGCs, respectively, and may represent one of numerous signaling pathways that play a role in determining ipsilateral versus contralateral RGC identity. Finally, we have confirmed the expression of several genes that mark “immaturity” in both ipsilateral RGCs and cells within proliferative zones of the retina. These findings suggest that the ontogeny of ipsilateral and contralateral RGCs and the mechanisms that regulate their differentiation are more diverse than previously expected.

### Ipsilateral and contralateral RGCs are molecularly distinct

Our gene profiling experiments reveal that ipsilateral and contralateral RGCs have distinct molecular signatures and can be distinguished during development not only by their axon guidance programs but also by many genes uncovered in our screen that play diverse functions in growth, differentiation, and fate specification.

To date, the only known specific markers of contralateral RGCs are the LIM homeodomain transcription factor Islet2 and the Pou-domain transcription factor Brn3a (Pou4f1). However, loss of Brn3a does not influence RGC axon laterality (Quina et al., 2005), and whereas Islet2 is important for contralaterality in late-born VT RGCs, it is not required for the major contralateral projection from extra-VT retina (Pak et al., 2004). Moreover, Islet2 is expressed in only a subset of contralateral RGCs. Thus, a transcription factor that specifies contralateral identity throughout the retina has not yet been identified. Our microarray study has revealed a number of different transcription factors that may fill such a role as a transcriptional regulator of contralateral RGC identity.

Together, Igf1, Fgf12, Tbx20, and Sema3e represent four new contralateral RGC markers during the peak period of RGC axon outgrowth. None of these genes have been studied in the context of RGC development. Tbx20 functions in heart development (Plageman and Yutzey, 2005) and cranial motor neuron migration (Song et al., 2006). The expression and guidance roles of semaphorin family members have been demonstrated at multiple points along the path of retinal axons (Callander et al., 2007; Kuwajima et al., 2012). Sema3e is known to be expressed at the optic chiasm (Sakai and Halloran, 2006), where it influences midline crossing decisions in the fish optic chiasm. Here, we show that it is also expressed in a subset of contralateral RGCs, raising the question of why this repulsive guidance cue is also expressed by RGCs. One possible explanation is the demonstration that RGC-secreted Sema3e regulates retinal angiogenesis through interactions with Plexin-D1 expressed on endothelial cells of sprouting blood vessels (Callander et al., 2007). However, whether Sema3e has a further function in regulating contralateral RGC axon growth and guidance has not been explored.

Furthermore, the expression of Tbx20 and Sema3e in a mosaic pattern in central retina suggests that both genes may mark a functional subtype of contralateral RGCs that is present only in central retina or in a subset that arises later in development. Alternatively, Tbx20 and Sema3e expression may be delayed in RGCs and thus only seen in the oldest RGCs present at this age, with those in central retina being the first to develop. Consistent with both of these explanations, both Sema3e and Tbx20 are not yet expressed in RGCs at E13.5 (data not shown).

We have also found that Igf1 and Fgf12 are upregulated in contralateral RGCs. The Igf and Fgf signaling pathways have broad mitogenic and cell survival effects during development, tissue repair, and tumor growth. Interestingly, unlike most secreted FGF family members, Fgf12 localizes to the nucleus (Smallwood et al., 1996), and thus may play a cell-autonomous role in RGCs. Igf1 is enriched in contralateral RGCs, whereas Igfbp5 is enriched in ipsilateral RGCs. Further suggesting the complementary expression pattern of these two genes, the most peripheral DT RGCs that lack Igf1 expression, express Igfbp5. However, because this observation was made correlatively by comparing ISH for these two genes on different retinal sections, definitive demonstration of mutually exclusive expression would require double-ISH within the same sections over different developmental time points. Although Igfbp5 is the only Igf binding protein detected by this microarray study, another Igf peptide, Igf2, was enriched in the ipsilateral RGC population. Igf2 has been previously reported as a gene preferentially expressed in peripheral mouse retina (Trimarchi et al., 2009).

Igf1 has also been implicated in neuronal circuitry formation by promoting axon outgrowth in corticospinal motor neurons (Ozdinler and Macklis, 2006) and by acting as a chemoattractant that directs olfactory neuron axons to innervate the lateral olfactory bulb (Scolnick et al., 2008). Insulin receptor signaling has also been implicated in synapse maturation and density within the *Xenopus* retinotectal circuit (Chiu et al., 2008). More recently, Igf1 has also been shown to promote αRGC-specific regeneration after axotomy (Duan et al., 2015). With its expression of multiple Igf signaling pathway proteins in distinct RGC populations, the developing retina offers an excellent opportunity to study the roles of these genes in neuronal development: in particular, the function of the complimentary genes Igf1 and Igfbp5 in defining the ipsilateral and contralateral RGC population or sectors of the retina.

### Ipsilateral RGCs are developmentally less mature than contralateral RGCs

One highlight of our gene profiling results is that ipsilateral but not contralateral RGCs express multiple transcription factors, such as Math5 and Sox2, known to be expressed in retinal progenitor cells (Xie et al., 2014). Two possible explanations for these observations are that (1) Zic2^+^ RGCs are developmentally less mature than their contralateral counterparts, and failure to downregulate these genes reflect their immaturity, and (2) these progenitor cell markers have additional functions in postmitotic Zic2^+^ RGCs and neuronal subtype identity and function. Indeed, several recent studies suggest that neural progenitor genes also have distinct functional roles in postmitotic neurons. Expression of early eye field transcription factors has been shown in both retinal progenitors and differentiated retinal cell types. For example, Pax6 is expressed in both retinal progenitors and RGCs and has a functional role in postmitotic RGC axonal guidance (Hsieh and Yang, 2009; Sebastián-Serrano et al., 2012). Similarly, although Sox2 is traditionally thought to maintain neural progenitor identity (Graham et al., 2003), it has also been demonstrated to play a role in neuronal differentiation (Cavallaro et al., 2008, Heavner et al., 2014). In the retina, complete *Sox2* ablation leads to dramatic loss of neural progenitors; however, reduction of Sox2 expression in hypomorphic or null compound heterozygotes leads to maturation defects specific to RGCs, whereas other cell types are mostly unaffected (Taranova et al., 2006).

Expression of progenitor genes in ipsilateral RGCs can be caused by perdurance of progenitor mRNAs in postmitotic RGCs and may simply reflect the relative neoteny of ipsilateral compared with contralateral RGCs. Alternatively, this expression may suggest that ipsilateral RGCs derive from a progenitor pool at a different competence, or differentiation, stage. Thus, the temporal control of neuronal differentiation may be a specific feature or regulator of ipsilateral versus contralateral RGC fate. In this vein, the same VT region that gives rise to ipsilateral RGCs during earlier retinal growth switches to generating contralaterally projecting RGCs after E16. The importance of timing in controlling fate specification of cell class in the retina (Livesey and Cepko, 2001) and elsewhere in the CNS (Caviness et al., 2003), as well as divergent of the same subclass (Imamura et al., 2011), supports this hypothesis.

### Cell cycle regulation in control of RGC subtype differentiation: a role for cyclin D2?

In support of the preponderance of early genes expressed in ipsilateral RGCs and ventrotemporal retina, we found that cyclin D2 is enriched in ventral retina, although not exclusively expressed there. One potential mechanism for controlling the timing of cell cycle exit and differentiation is through differential expression of cell cycle regulators (Dyer and Cepko, 2001; Ross, 2011). Thus, the finding that cyclin D2 is expressed within the VT RGC zone is particularly interesting. Cyclin D1 is the G1 cyclin expressed throughout most of retinal development (Sicinski et al., 1995). Although the expression of cyclin D2 in the peripheral margin of the retina has been previously shown as part of large-scale expression analyses (Glickstein et al., 2007; Trimarchi et al., 2009), none of those studies dissected out the changes in cyclin D2 expression along the dorsoventral and nasotemporal axes. Unlike the microphthalmia seen in the cyclin D1–null mouse (Fantl et al., 1995; Sicinski et al., 1995), no gross retinal phenotype has been identified in the cyclin D2–null mouse (Sicinski et al., 1996). Studies in cortical, cerebellar, and spinal cord development have demonstrated a cell type–specific dependence on the different cyclin D family members, in particular cyclin D1 versus D2 (Huard et al., 1999; Lukaszewicz and Anderson, 2011; Ross, 2011; Petros et al., 2015). Thus, the distinct expression patterns of cyclin D1 and D2 in retinal development, and the close juxtaposition of cyclin D2 with the highly specialized subpopulation of RGCs projecting ipsilaterally, hint at a potential function of cyclin D2 expression in ipsilateral RGC production. In support of this, we observed an intermingling of cyclin D2^+^ cells with Brn3^+^ RGCs at E13.5 and E14.5 (Fig. 7*B*, *C*, yellow arrows) within ventral but not dorsal retina, where the two expression domains have distinct boundaries. This overlap in the cyclin D2 and RGC domains raises the possibility that the Ccnd2^+^ cells within this overlapping domain are in the process of differentiating into Brn3^+^ RGCs. Importantly, asymmetry in cyclin D2 expression is apparent only from E11.5 to E15.5, before or during the period when the majority of ipsilateral RGCs are produced, and no longer at E16.5, when ipsilateral RGC production wanes. Thus, the tightly regulated spatiotemporal expression pattern of cyclin D2 within the murine retina is highly suggestive of a population of cyclin D2–expressing progenitors that gives rise to ipsilateral RGCs. These possibilities are being addressed by ongoing studies in our laboratory.

Prior studies in the albino mouse model have shown that changes in the timing and rate of cell production within the ventrotemporal retina lead to a decrease in ipsilateral RGC production (Bhansali et al., 2014). However, the molecular pathways that regulate this rate remain to be determined. The concerted action of cyclin D1 and D2 on cell cycle exit and length could confer one such regulatory mechanism.

### Future perspectives

The developmental roles of the many genes differentially expressed in ipsilateral and contralateral RGCs will be revealed only through further gain- and loss-of-function experiments, through the use of mutant mouse models or overexpression and knockdown studies by *in utero* and *ex vivo* electroporation (Petros et al., 2009a). However, the transcriptomic data and *in vivo* expression studies we present themselves provide novel insight into the development of ipsilateral and contralateral RGCs and the mechanisms through which neuronal diversification can be accomplished in the retina. Additional studies using similar retrograde labeling and cell sorting approaches to isolate ipsilateral and contralateral RGCs in mature retina can further elucidate how these two cell types are molecularly and functionally distinct beyond development, after injury, and in regeneration. Moreover, our use of rapid retrograde labeling *ex vivo* to isolate projection neurons that have not yet reached their targets can be applied to the purification of other neuronal subtypes during embryonic ages.

## Synthesis

Reviewing Editor: Esther Stoeckli, University of Zurich Decisions are customarily a result of the Reviewing Editor and the peer reviewers coming together and discussing their recommendations until a consensus is reached. When revisions are invited, a fact-based synthesis statement explaining their decision and outlining what is needed to prepare a revision will be listed below. The following reviewer(s) agreed to reveal their identity: Robert Hindges, Harald Hutter.

In this manuscript the authors describe a differential expression analysis identifying genes enriched in either ipsilaterally or contralaterally projecting RGCs. They confirm their data with qPCR experiments and characterize the retinal expression of selected genes using in situ hybridization and antibody staining. The study identifies a large number of differentially expressed genes and provides a starting point for a functional characterization of those genes. The experiments are well documented and support the conclusions.

I agree with the reviewers' conclusions that the screen provides an interesting starting point for future studies. The method used in this study to identify ipsi- and contralaterally projecting RGCs by dextran labeling allows for the unequivocal selection of the respective cell populations as a source for differentially expressed genes. However, I also share their concerns that the validity of the approach has suffered from the fact that the two pools of cells (ipsi- versus contra) have not been treated in the same manner. This fact likely distorts the relative abundance of differentially expressed genes. Still we feel that the data provide an important resource for others interested in (the function of) differentially expressed genes in the retina. Therefore, we suggest that you comment on this caveat in the manuscript along the lines suggested by reviewer 1.

Specific comments made by reviewer 1:

In general, the work could provide a useful list of different markers that are differentially expressed between these cell populations. However, I have a concern regarding the set up of the experiments. The authors state that they had to amplify the cDNA from the ipsi population, due to the low numbers of cells recovered. Does that mean no amplification was carried out for the contra population? If this is the case, the two samples (ipsi vs contra) are not comparable without problems, due to the different preparations. Although some of the known markers for ipsilateral-projecting RGC are correctly found in the dataset, it could be that many others in the list are not real. This could also be a possible reason why so many specific genes have been found for the ipsi population in comparison to the relatively low number for the contra population. A possible approach would be to reduce the number of contralateral projecting RGCs (maybe from the peripheral region, to "correct" for the different developmental stages between central and peripheral RGCs) and do similar amplification protocols for all samples.

The expression images for Sema3e and Igf1 show also reduced/absent expression in the dorsal periphery. The authors explain this by suggesting that these RGCs have not yet accumulated as much of the transcript as the more central RGCs. Could it be that this also is true for the peripheral RGCs in the ventral retina?

The authors suggest a complementary role (and expression domain) of Igfbp5 and Igf1. However, from the images shown it looks like that the Igf1 positive area in the periphery is much larger than the region positive for Igfbp5. Have the authors tried to do two-color ISH for these markers to show their expression domains in the same tissue? If this is difficult, maybe adjacent sections for the two different probes could help.

The authors show that Igfbp5 is reduced in VT retina of Foxd1 knock out mice. However, they do not show what happens to the DT expression domain. This should be included as a control to show the specificity for the ipsilateral RGCs.

The authors describe an asymmetrical expression of Ccnd2 between dorsal and ventral retina. However this is very difficult to see in the ISH image given.

Specific comments by reviewer 2:

page 13/14

"In total, 299 genes (404 probe sets) were found to be enriched in ipsilateral RGCs and 40 genes (47 probe sets) in contralateral RGCs, using a 2-fold cutoff (Fig. 2), suggesting that an extensive genetic program is activated to generate ipsilateral RGCs."

Comment

The authors seem to suggest here that ipsilateral cells are more distinct than contralateral cells in their expression profile (299 upregulated genes versus 40 genes). However the authors concede that they might have missed an (unknown) number of contralateral genes (they mention Islet2 as an example), because contralateral cells are heterogeneous (e.g. Islet2 is expressed only in a subset of contralateral cells). It is therefore not clear to me, whether the observed difference (299 versus 40 genes) is meaningful. The authors should make it more explicitly clear that contralateral RGCs are heterogeneous in their expression profiles, which leads to an underestimation of contralateral enriched genes.

page 15:

"Of the 7 new contralateral genes tested, the following genes showed exclusive expression in contralateral RGCs (Islet1/2+Zic2- cells)"

The authors should comment on why these 7 genes were selected for further analysis. It looks like an arbitrary selection.

page 20

"Cyclin D2+ cells that showed positive staining for Zic2 and Islet1/2 were very rare,"

According to earlier statements by the authors the cells used for expression profiling express both Islet2 and Zic2. The authors find Cyclin D2 enriched in those cells by microarray analysis. I would therefore expect that immunostainings of retina show Cyclin D2 co-expressed with Zic2 and Islet2. Apparently this is not the case (see quoted statement above). I find this rather puzzling. The authors should comment on this.

According to the microarray data, Cyclin D1 is also enriched in ipsilateral cells (3.88 FC, see table 2-3). However the authors claim that: "In contrast to cyclin D2, cyclin D1 expression is homogeneous throughout proliferating regions in the retina (not shown)." This seems to contradict the enrichment seen in the microarray experiments. The authors should comment on this.

Table 1:

There is no good correlation between microarray and qPCR data with respect to fold-change (FC). E.g. zic1 and zic2 show a similar FC in microarray data but very different FCs in qPCR experiments (6 for Zic1 versus 64 for Zic2). The authors should discuss this discrepancy.
